# Supplementary figures and images for: IFN alpha inducible protein 27 (IFI27) acts as a positive regulator of PACT-dependent PKR activation after RNA virus infections
Source: PLoS Pathog. 2025 Jun 16;21(6):e1013246. doi: 10.1371/journal.ppat.1013246 (PMC12204625; doi:10.1371/journal.ppat.1013246)

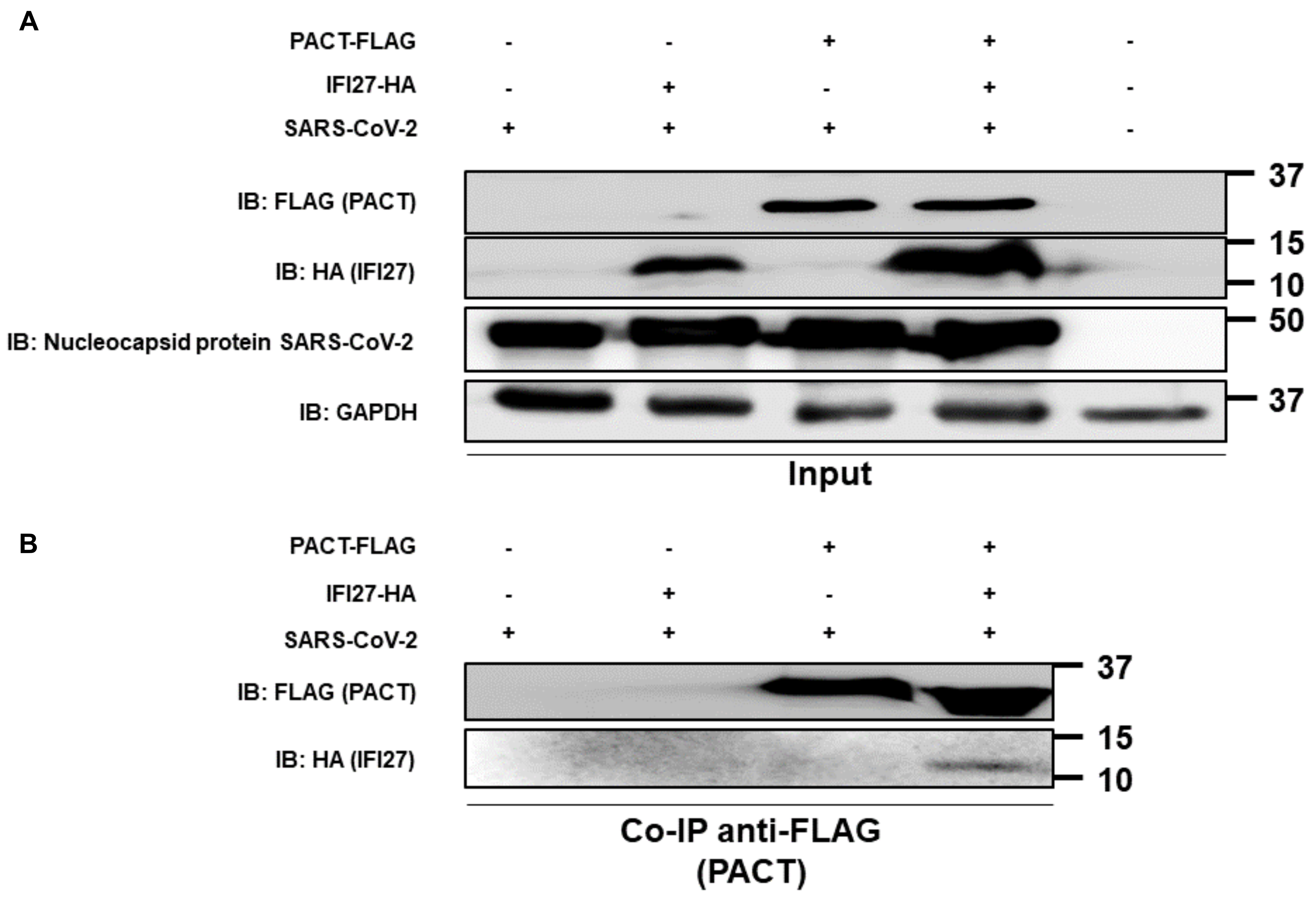

Supplement: S1 Fig — (A and B) HEK-293T-hACE2 cells were transiently transfected either with a pCAGGS-PACT-FLAG plasmid alone, a pCAGGS IFI27-HA plasmid alone, both pCAGGS-PACT-FLAG and pCAGGS-IFI27-HA plasmids, or an emtpy pCAGGS plasmid (Empty) and 24h later, transfected cells were infected with SARS-CoV-2 at a multiplicity of infection (MOI) of 0.5 during 24 hours. At 24h after infection, protein extracts were obtained by lysis. (B) Protein extracts were analysed by Western blot (input) with antibodies specific for FLAG (to detect PACT-FLAG), HA (to detect IFI27-HA), SARS-CoV-2 nucleocapsid protein, and GAPDH. Molecular weights are indicated on the right of the panels (in kilodaltons). (B) Protein extracts were incubated with FLAG-bound agarose beads to retain PACT-FLAG and all its associated proteins. Eluates were analysed by Western blot, to detect PACT-FLAG and IFI27-HA by using anti-FLAG (to detect PACT-FLAG, top panel) and anti-HA (to detect IFI27-HA, bottom panel) antibodies after the Co-IP. Molecular weights are indicated on the right of the panels (in kilodaltons). (TIF) [file ppat.1013246.s001.tif]

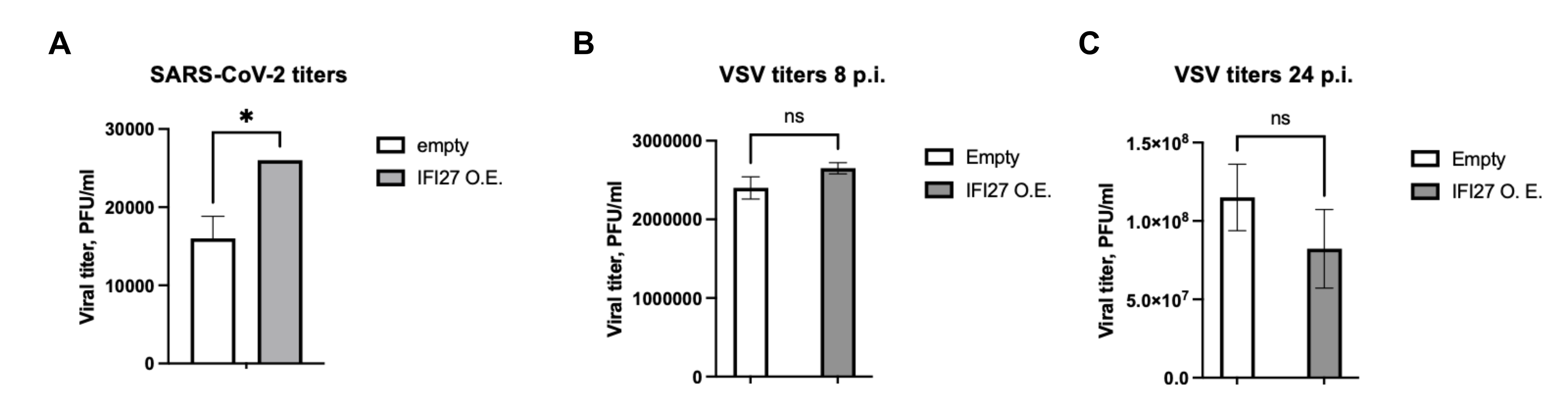

Supplement: S2 Fig — (A, B, C) HEK-293T cells were transiently transfected with a pCAGGS-IFI27-HA plasmid (IFI27 O.E.) or an emtpy pCAGGS plasmid (Empty) and 24h later, transfected cells were left mock-infected or infected with SARS-CoV-2 at a multiplicity of infection (MOI) of 1 during 24 hours (A), or infected with VSV at a MOI of 1 during 8 (B) or 24 hours (C). SARS-CoV-2 and VSV viral titers were determined by plaque assay (plaque forming units, PFU/ml) in confluent monolayers of Vero E6 cells seeded in 24-well plates, as previously described [43,44]. Data is represented as the mean and standard deviations of triplicate measures. ns (non-significant) p > 0.05, *p < 0.05, **p < 0.01, ***p < 0.001, **** p < 0.0001 (for comparisons using unpaired two-tailed Student’s t test in A, B and C). (TIF) [file ppat.1013246.s002.tif]

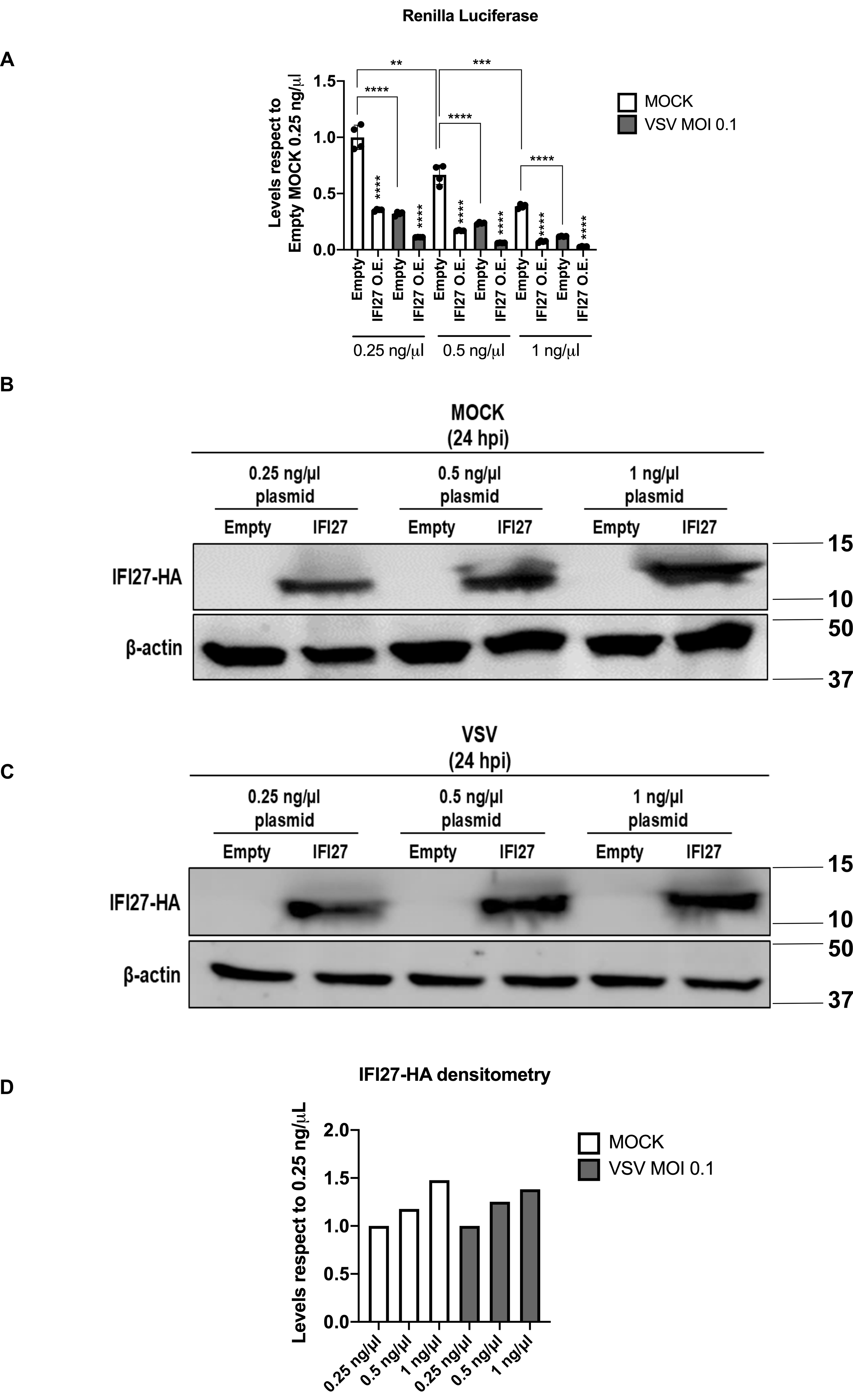

Supplement: S3 Fig — (A, B, C, D) HEK-293T cells were transiently transfected with a pCAGGS-IFI27-HA plasmid (IFI27 O.E.) or an emtpy pCAGGS plasmid (Empty) together with a pRL plasmid expressing RLuc luciferase, and 24h later, transfected cells were left mock-infected (A, B, D) or infected with VSV (A, C, D) at a multiplicity of infection (MOI) of 0.1 during 24 hours. 24 hpi, protein extracts were obtained, and RLuc luminiscence was measured (A). These protein extracts were also used to measure protein levels of IFI27-HA and β-actin by Western blot employing their respective antibodies. Molecular weight is indicated on the right of the panels (in kilodaltons). Western blots were quantified by densitometry using ImageJ software. The amount of IFI27-HA was normalised to the amounts of β-actin (results of quantification showed in bars below the immunoblots, with levels relative to MOCK 0.25 ng/µl). Data is represented as the means and standard deviations of triplicate measures. p > 0.05, *p < 0.05, **p < 0.01, ***p < 0.001, **** p < 0.0001 (for comparisons using unpaired two-tailed Student’s t test in A). The asterisks above each bar represent the comparison vs. the control condition. (TIF) [file ppat.1013246.s003.tif]

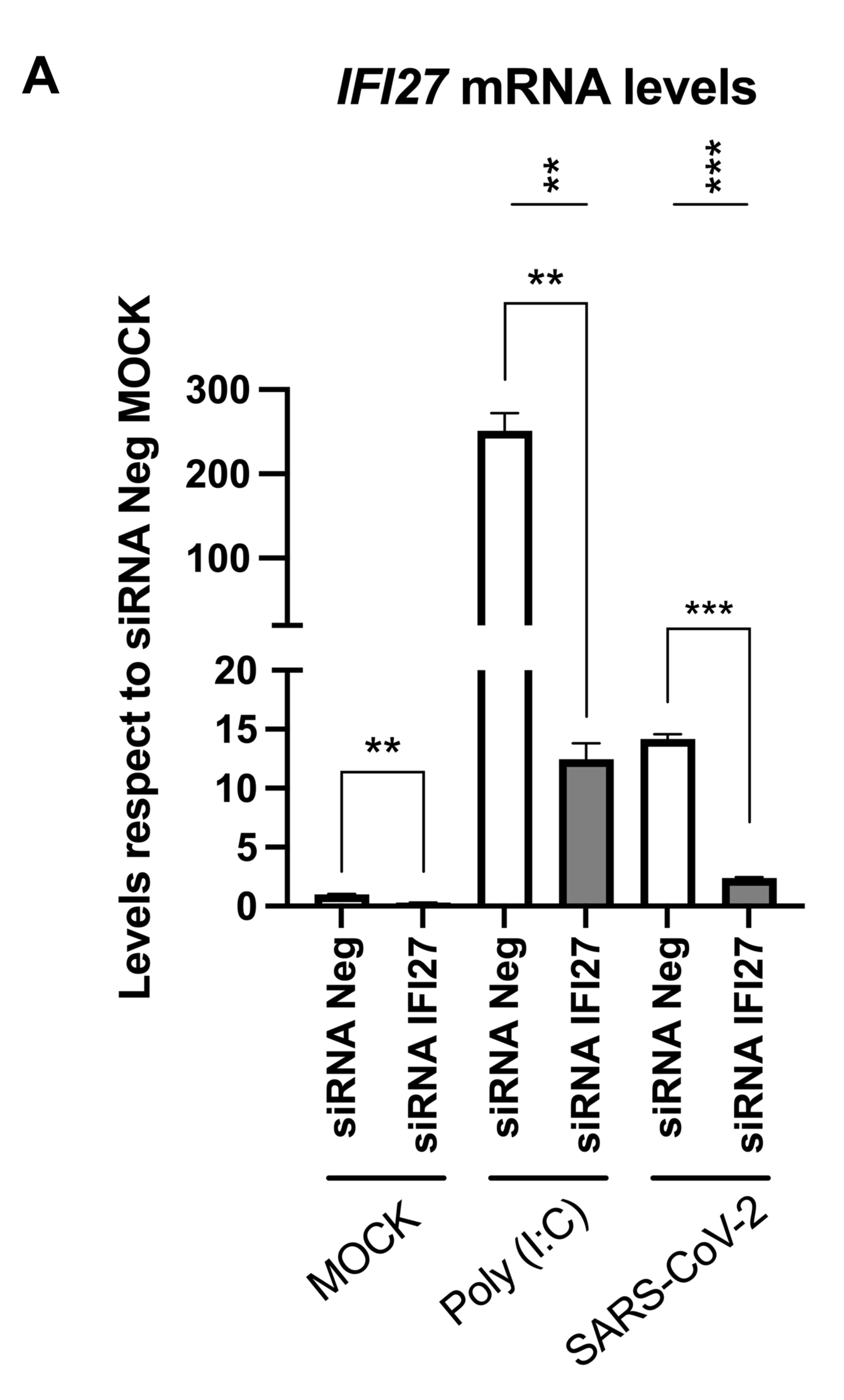

Supplement: S4 Fig — A549-hACE2 WT cells were transfected twice, with a 24-hour gap between each transfection, either with a negative control non-targeting siRNA (siRNA Neg) or with an IFI27 siRNA (siRNA IFI27). 24 hours after the second transfection, cells were either mock or SARS-CoV-2 infected at a MOI of 1 for 24 hours. Total RNA was extracted, and a qRT-PCR was performed, to determine the level of expression of the IFI27 mRNA in each condition, comparing siRNA Neg and siRNA IFI27 effect on IFI27 expression. Data is represented as the mean and standard deviations of triplicate measures. ns (non-significant), p > 0.05, *p < 0.05, **p < 0.01, ***p < 0.001, **** p < 0.0001 (for comparisons using unpaired two-tailed Student’s t test). (TIF) [file ppat.1013246.s004.tif]

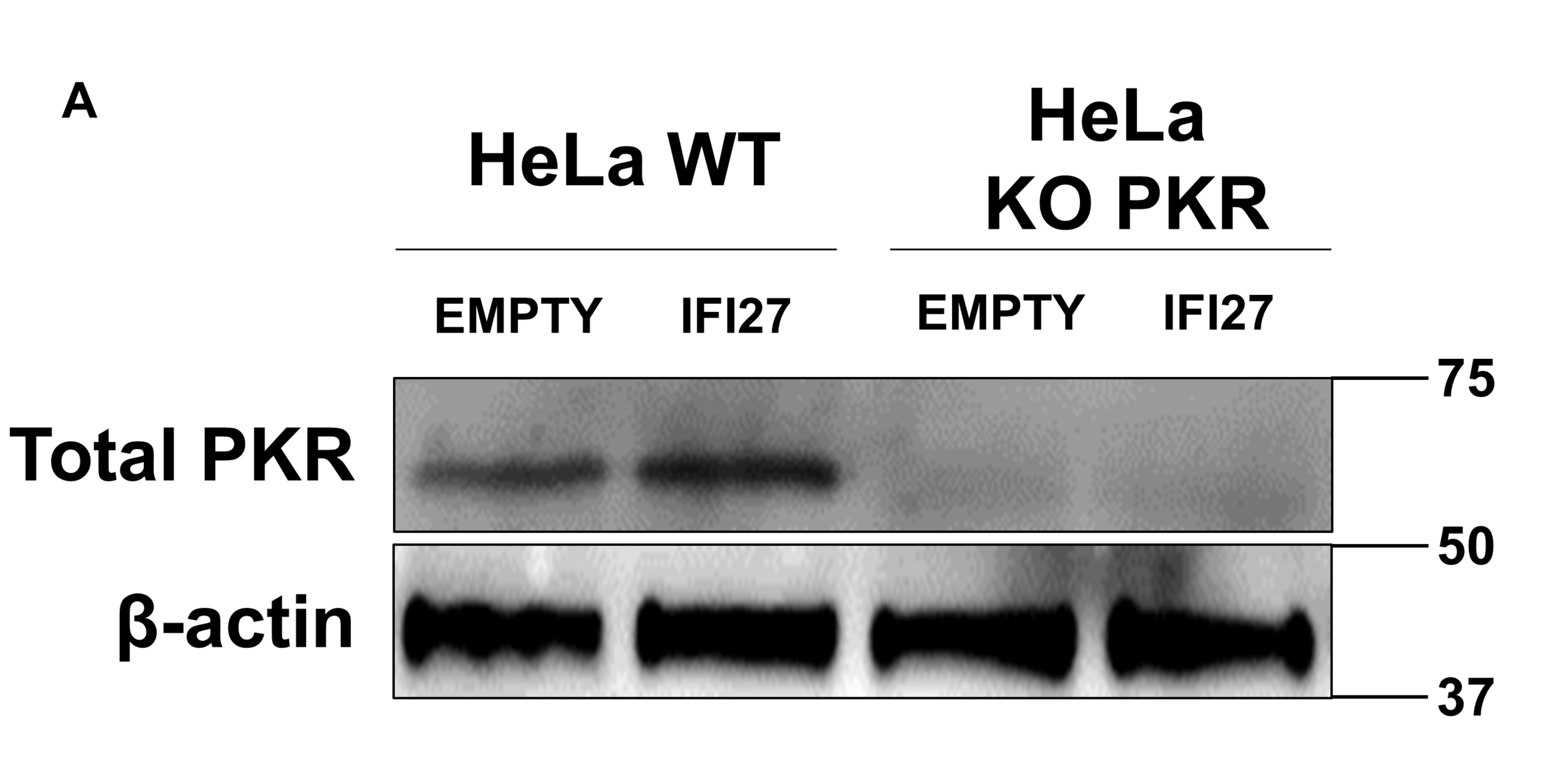

Supplement: S5 Fig — HeLa WT and HeLa PKR KO cells were transiently transfected with a pCAGGS-IFI27-HA plasmid (IFI27 O.E.) or an empty pCAGGS plasmid (empty). 24 hours post-transfection, protein extracts were obtained by lysis and the protein levels of total PKR and β-actin were measured by Western blot employing their respective antibodies. Molecular weight is indicated on the right of the panels (in kilodaltons). (TIF) [file ppat.1013246.s005.tif]

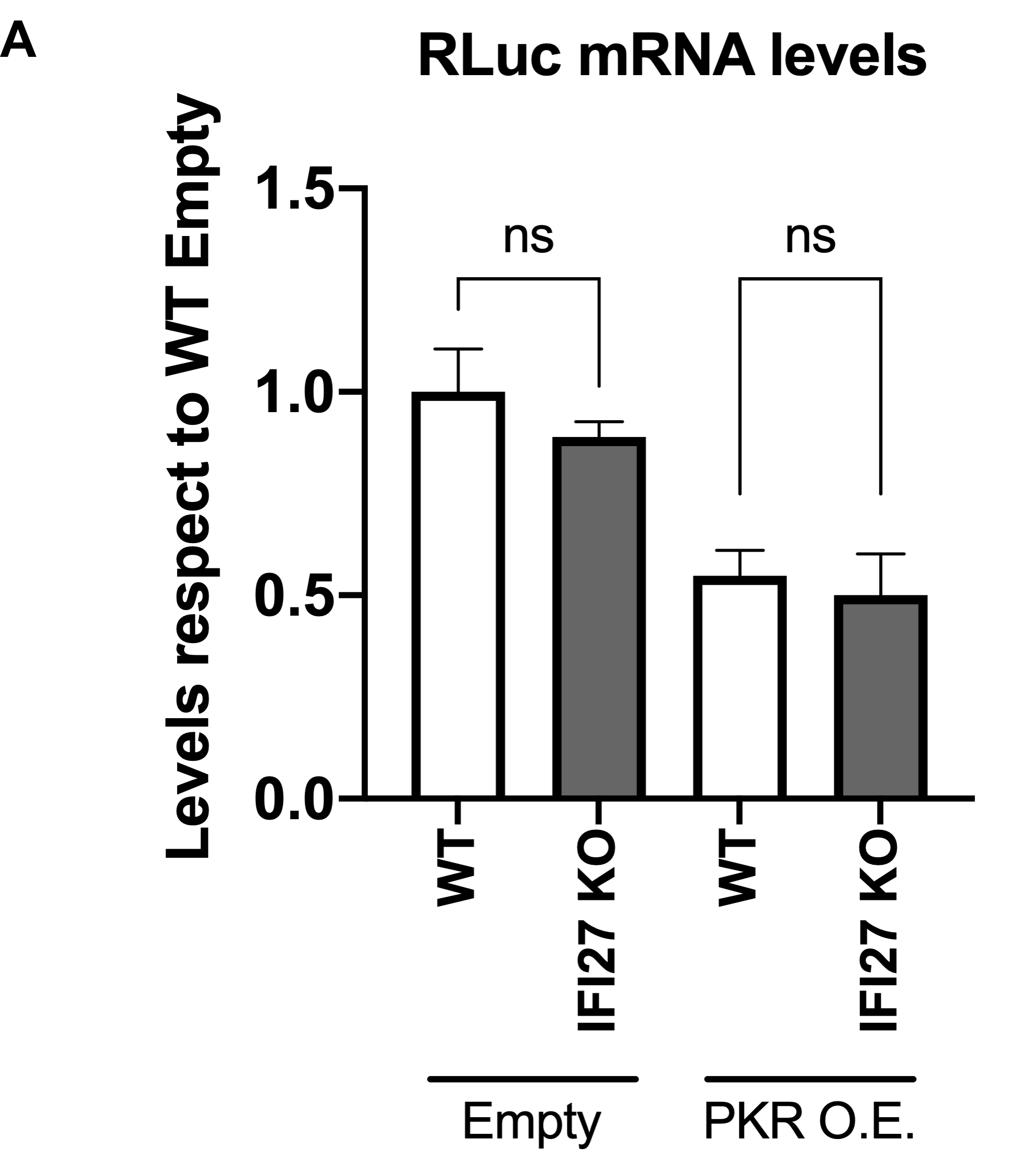

Supplement: S6 Fig — A549-hACE2 WT and A549-hACE2 IFI27 KO cells were transiently transfected with a pCAGGS-PKR-myc plasmid (PKR O.E.) or an Empty pCAGGS plasmid (Empty) in combination with an Rluc expressing pRL plasmid. At 24h later, the cellular RNAs were purified and the levels of RLuc mRNAs were measured by RT-qPCR. Data are represented as the mean and standard deviations of triplicate measures. ns (non-significant). (TIF) [file ppat.1013246.s006.tif]
