# Supplementary material for: IFN alpha inducible protein 27 (IFI27) acts as a positive regulator of PACT-dependent PKR activation after RNA virus infections
Source: PLoS Pathog. 2025 Jun 16;21(6):e1013246. doi: 10.1371/journal.ppat.1013246 (PMC12204625; doi:10.1371/journal.ppat.1013246)
Supplement: S1 Table — HEK-293T cells were transfected with a pCAGGS plasmid encoding for IFI27 fused to HA tag (pCAGGS-IFI27-HA) or with a pCAGGS empty plasmid, as control. 24 hours later, cells were treated with Poly(I:C) at 3000 ng/ml. At 24h after poly(I:C) transfection, protein extracts were obtained by lysis and were incubated with HA-bound agarose beads to retain IFI27-HA and all its associated proteins, which were then identified by MS. The proteins identified only in the IFI27-HA-overexpressing cells are shown. Protein accession number (prot_acc), protein description (prot_desc), prot_score, prot_mass, prot_matches_sig, prot_sequences_sig, prot_coverage and prot_PI (isoelectric point) are indicated, being these terms previously explained in (https://www.matrixscience.com/help/csv_headers.html). (DOCX) [file ppat.1013246.s007.docx]

| **prot_acc** | **prot_desc** | **prot_score** | **prot_mass** | **prot_matches_sig** | **prot_sequences_sig** | **prot_cover** | **prot_pi** |
| --- | --- | --- | --- | --- | --- | --- | --- |
| Q7Z406 | Myosin-14 OS=Homo sapiens GN=MYH14 PE=1 SV=2 | 1554 | 228514 | 37 | 29 | 16 | 5,52 |
| O95793 | Double-stranded RNA-binding protein Staufen homolog 1 OS=Homo sapiens GN=STAU1 PE=1 SV=2 | 925 | 63373 | 20 | 17 | 36,7 | 9,46 |
| O94832 | Unconventional myosin-Id OS=Homo sapiens GN=MYO1D PE=1 SV=2 | 889 | 116772 | 19 | 19 | 21,6 | 9,44 |
| F5H6E2 | Unconventional myosin-Ic OS=Homo sapiens GN=MYO1C PE=1 SV=1 | 881 | 119564 | 17 | 17 | 21,7 | 9,46 |
| A0A0A0MRM8 | Unconventional myosin-VI OS=Homo sapiens GN=MYO6 PE=1 SV=1 | 842 | 146028 | 15 | 15 | 17,5 | 8,78 |
| E9PDF6 | Unconventional myosin-Ib OS=Homo sapiens GN=MYO1B PE=1 SV=1 | 835 | 129228 | 18 | 17 | 18,5 | 9,36 |
| G8JLA2 | Myosin light polypeptide 6 OS=Homo sapiens GN=MYL6 PE=1 SV=1 | 651 | 17216 | 18 | 10 | 75,7 | 4,46 |
| A0A1X7SBS1 | Heterogeneous nuclear ribonucleoprotein U OS=Homo sapiens GN=HNRNPU PE=1 SV=1 | 623 | 82296 | 12 | 12 | 20,7 | 5,64 |
| P11142 | Heat shock cognate 71 kDa protein OS=Homo sapiens GN=HSPA8 PE=1 SV=1 | 606 | 71038 | 12 | 11 | 23,5 | 5,37 |
| P19525 | Interferon-induced, double-stranded RNA-activated protein kinase OS=Homo sapiens GN=EIF2AK2 PE=1 SV=2 | 565 | 62424 | 11 | 11 | 26 | 8,58 |
| Q15366 | Poly(rC)-binding protein 2 OS=Homo sapiens GN=PCBP2 PE=1 SV=1 | 553 | 38878 | 13 | 9 | 45,2 | 6,33 |
| P68032 | Actin, alpha cardiac muscle 1 OS=Homo sapiens GN=ACTC1 PE=1 SV=1 | 544 | 42268 | 58 | 11 | 28,6 | 5,23 |
| A0A087WWU8 | Tropomyosin alpha-3 chain OS=Homo sapiens GN=TPM3 PE=1 SV=1 | 511 | 26588 | 10 | 10 | 39,6 | 4,75 |
| P52907 | F-actin-capping protein subunit alpha-1 OS=Homo sapiens GN=CAPZA1 PE=1 SV=3 | 511 | 33040 | 9 | 9 | 45,1 | 5,45 |
| Q5T6W2 | Heterogeneous nuclear ribonucleoprotein K (Fragment) OS=Homo sapiens GN=HNRNPK PE=1 SV=1 | 508 | 41965 | 11 | 11 | 35,9 | 5,43 |
| A0A0A0MTC4 | Double-stranded RNA-binding protein Staufen homolog 2 OS=Homo sapiens GN=STAU2 PE=1 SV=1 | 505 | 43343 | 8 | 8 | 31,9 | 9,44 |
| P14649 | Myosin light chain 6B OS=Homo sapiens GN=MYL6B PE=1 SV=1 | 495 | 22842 | 13 | 10 | 48,1 | 5,56 |
| O75569 | Interferon-inducible double-stranded RNA-dependent protein kinase activator A OS=Homo sapiens GN=PRKRA PE=1 SV=1 | 494 | 34750 | 10 | 10 | 44,4 | 8,69 |
| O77727 | Keratin, type I cytoskeletal 15 (Contact-Cont) OS=Ovis aries GN=KRT15 PE=2 SV=1 | 490 | 48740 | 32 | 10 | 15,9 | 4,73 |
| Q9UHB6 | LIM domain and actin-binding protein 1 OS=Homo sapiens GN=LIMA1 PE=1 SV=1 | 480 | 85541 | 10 | 10 | 20,2 | 6,41 |
| Q9NYL9 | Tropomodulin-3 OS=Homo sapiens GN=TMOD3 PE=1 SV=1 | 427 | 39708 | 8 | 8 | 29,3 | 5,08 |
| A0A087WZR1 | Interferon alpha-inducible protein 27, mitochondrial OS=Homo sapiens GN=IFI27 PE=1 SV=1 | 400 | 11233 | 15 | 5 | 67,2 | 11,07 |
| P32969 | 60S ribosomal protein L9 OS=Homo sapiens GN=RPL9 PE=1 SV=1 | 387 | 21942 | 7 | 7 | 53,6 | 9,96 |
| Q15633 | RISC-loading complex subunit TARBP2 OS=Homo sapiens GN=TARBP2 PE=1 SV=3 | 373 | 39521 | 6 | 6 | 22,4 | 6,11 |
| H0YK48 | Tropomyosin alpha-1 chain OS=Homo sapiens GN=TPM1 PE=1 SV=1 | 372 | 28609 | 7 | 7 | 26,2 | 4,74 |
| Q16643 | Drebrin OS=Homo sapiens GN=DBN1 PE=1 SV=4 | 369 | 71753 | 7 | 7 | 16 | 4,41 |
| B1AK87 | Capping protein (Actin filament) muscle Z-line, beta, isoform CRA_a OS=Homo sapiens GN=CAPZB PE=1 SV=1 | 363 | 29507 | 7 | 7 | 29,2 | 6,45 |
| P67936 | Tropomyosin alpha-4 chain OS=Homo sapiens GN=TPM4 PE=1 SV=3 | 346 | 28596 | 6 | 6 | 21,8 | 4,67 |
| H0Y6E7 | RNA-binding motif protein, X chromosome (Fragment) OS=Homo sapiens GN=RBMX PE=1 SV=2 | 342 | 31837 | 7 | 6 | 21,6 | 9,84 |
| B7Z645 | Heterogeneous nuclear ribonucleoprotein Q OS=Homo sapiens GN=SYNCRIP PE=1 SV=1 | 338 | 52152 | 6 | 6 | 14 | 9,19 |
| F8VR82 | Serine/threonine-protein phosphatase OS=Homo sapiens GN=PPP1CC PE=1 SV=1 | 279 | 31610 | 5 | 5 | 25,2 | 5,57 |
| H0YKX5 | Tropomyosin alpha-1 chain (Fragment) OS=Homo sapiens GN=TPM1 PE=1 SV=1 | 277 | 16401 | 6 | 6 | 31,7 | 4,7 |
| A0A087WXM6 | 60S ribosomal protein L17 (Fragment) OS=Homo sapiens GN=RPL17 PE=3 SV=1 | 271 | 19757 | 7 | 6 | 49,1 | 10,05 |
| H0YKS4 | Annexin (Fragment) OS=Homo sapiens GN=ANXA2 PE=1 SV=1 | 266 | 19610 | 5 | 5 | 38,6 | 5,68 |
| I3L3P7 | 40S ribosomal protein S15a OS=Homo sapiens GN=RPS15A PE=1 SV=1 | 266 | 11562 | 6 | 6 | 53 | 10,18 |
| D6RJ07 | Zinc finger protein 346 OS=Homo sapiens GN=ZNF346 PE=1 SV=1 | 264 | 35129 | 4 | 4 | 24,5 | 9,34 |
| A0A0C4DGB6 | Serum albumin OS=Homo sapiens GN=ALB PE=1 SV=1 | 261 | 70791 | 6 | 6 | 10,3 | 5,99 |
| I3L1L3 | Myb-binding protein 1A (Fragment) OS=Homo sapiens GN=MYBBP1A PE=1 SV=1 | 260 | 140937 | 5 | 5 | 7,5 | 9,34 |
| M0R1A7 | 60S ribosomal protein L18a OS=Homo sapiens GN=RPL18A PE=1 SV=1 | 259 | 17604 | 7 | 6 | 40,1 | 10,89 |
| Q96SI9 | Spermatid perinuclear RNA-binding protein OS=Homo sapiens GN=STRBP PE=1 SV=1 | 227 | 74158 | 6 | 6 | 7,7 | 8,91 |
| E9PFP8 | Poly(rC)-binding protein 3 OS=Homo sapiens GN=PCBP3 PE=1 SV=1 | 222 | 38393 | 4 | 4 | 14,1 | 8,22 |
| P62280 | 40S ribosomal protein S11 OS=Homo sapiens GN=RPS11 PE=1 SV=3 | 193 | 18557 | 4 | 4 | 32,3 | 10,31 |
| A0A087WVQ9 | Elongation factor 1-alpha 1 OS=Homo sapiens GN=EEF1A1 PE=1 SV=1 | 191 | 48129 | 4 | 4 | 12,9 | 9,12 |
| Q6WCQ1 | Myosin phosphatase Rho-interacting protein OS=Homo sapiens GN=MPRIP PE=1 SV=3 | 184 | 117105 | 4 | 4 | 6,2 | 5,89 |
| O15145 | Actin-related protein 2/3 complex subunit 3 OS=Homo sapiens GN=ARPC3 PE=1 SV=3 | 173 | 20717 | 4 | 4 | 23,6 | 8,78 |
| H0YB22 | 40S ribosomal protein S14 (Fragment) OS=Homo sapiens GN=RPS14 PE=1 SV=1 | 157 | 13165 | 3 | 3 | 35 | 9,91 |
| F8WCF6 | Actin-related protein 2/3 complex subunit 4 OS=Homo sapiens GN=ARPC4-TTLL3 PE=3 SV=1 | 151 | 21137 | 4 | 4 | 21 | 8,89 |
| A0A087WY00 | Unconventional myosin-Va OS=Homo sapiens GN=MYO5A PE=1 SV=1 | 145 | 213447 | 3 | 3 | 1,8 | 8,82 |
| A0A0U1RR32 | Histone H2A OS=Homo sapiens GN=HIST1H3D PE=3 SV=1 | 144 | 18470 | 4 | 3 | 20,7 | 11,52 |
| P62241 | 40S ribosomal protein S8 OS=Homo sapiens GN=RPS8 PE=1 SV=2 | 139 | 24420 | 3 | 3 | 19,7 | 10,32 |
| P40939 | Trifunctional enzyme subunit alpha, mitochondrial OS=Homo sapiens GN=HADHA PE=1 SV=2 | 132 | 83545 | 3 | 3 | 7,2 | 9,16 |
| Q86SG5 | Protein S100-A7A OS=Homo sapiens GN=S100A7A PE=1 SV=3 | 124 | 11390 | 4 | 2 | 22,8 | 6,89 |
| H3BTH6 | F-box only protein 22 (Fragment) OS=Homo sapiens GN=FBXO22 PE=1 SV=1 | 121 | 26852 | 3 | 3 | 15,8 | 6,66 |
| A0A0D9SET8 | F-actin-capping protein subunit alpha-2 OS=Homo sapiens GN=CAPZA2 PE=1 SV=1 | 108 | 20390 | 1 | 1 | 16,1 | 6,07 |
| O75475 | PC4 and SFRS1-interacting protein OS=Homo sapiens GN=PSIP1 PE=1 SV=1 | 106 | 60159 | 2 | 2 | 8,1 | 9,15 |
| J3KQE5 | GTP-binding nuclear protein Ran (Fragment) OS=Homo sapiens GN=RAN PE=1 SV=1 | 101 | 26983 | 2 | 2 | 9 | 9,62 |
| A0A0D9SF53 | ATP-dependent RNA helicase DDX3X OS=Homo sapiens GN=DDX3X PE=1 SV=1 | 87 | 81978 | 2 | 2 | 3,1 | 8,29 |
| M0QZ52 | Calmodulin OS=Homo sapiens GN=CALM3 PE=1 SV=1 | 87 | 9348 | 1 | 1 | 36,1 | 3,89 |
| A2A2V2 | RNA-binding protein 34 (Fragment) OS=Homo sapiens GN=RBM34 PE=1 SV=1 | 84 | 45982 | 2 | 2 | 6,4 | 10,09 |
| C9JI87 | Voltage-dependent anion-selective channel protein 1 (Fragment) OS=Homo sapiens GN=VDAC1 PE=1 SV=6 | 80 | 20308 | 2 | 2 | 13,1 | 6,74 |
| O43143 | Pre-mRNA-splicing factor ATP-dependent RNA helicase DHX15 OS=Homo sapiens GN=DHX15 PE=1 SV=2 | 77 | 91519 | 1 | 1 | 1,5 | 7,12 |
| Q9ULJ8 | Neurabin-1 OS=Homo sapiens GN=PPP1R9A PE=1 SV=2 | 77 | 123497 | 2 | 2 | 1,6 | 5,01 |
| X6R631 | 39S ribosomal protein L55, mitochondrial OS=Homo sapiens GN=MRPL55 PE=1 SV=2 | 77 | 6845 | 1 | 1 | 29,1 | 9,3 |
| P27824 | Calnexin OS=Homo sapiens GN=CANX PE=1 SV=2 | 75 | 67894 | 3 | 3 | 6,8 | 4,47 |
| Q5SSJ5 | Heterochromatin protein 1-binding protein 3 OS=Homo sapiens GN=HP1BP3 PE=1 SV=1 | 73 | 61399 | 3 | 3 | 5,8 | 9,69 |
| Q9Y2Q9 | 28S ribosomal protein S28, mitochondrial OS=Homo sapiens GN=MRPS28 PE=1 SV=1 | 72 | 20968 | 1 | 1 | 10,2 | 9,21 |
| F5GXS2 | Alpha-actinin-4 OS=Homo sapiens GN=ACTN4 PE=1 SV=2 | 69 | 59757 | 2 | 2 | 4,2 | 4,82 |
| V9GYA7 | 28S ribosomal protein S29, mitochondrial (Fragment) OS=Homo sapiens GN=DAP3 PE=1 SV=1 | 68 | 23681 | 1 | 1 | 7,1 | 7,52 |
| A0A087X035 | Interferon alpha-inducible protein 27, mitochondrial (Fragment) OS=Homo sapiens GN=IFI27 PE=4 SV=1 | 63 | 12189 | 1 | 1 | 10 | 6,74 |
| A0A1B0GTT5 | Vimentin (Fragment) OS=Homo sapiens GN=VIM PE=1 SV=1 | 62 | 16415 | 1 | 1 | 6,7 | 10,26 |
| A8MUF7 | Hemoglobin subunit epsilon (Fragment) OS=Homo sapiens GN=HBE1 PE=1 SV=1 | 56 | 9464 | 1 | 1 | 11,5 | 9,1 |
| P56182 | Ribosomal RNA processing protein 1 homolog A OS=Homo sapiens GN=RRP1 PE=1 SV=1 | 56 | 52990 | 1 | 1 | 2,8 | 9,39 |
| M0QX71 | Glutamate-rich WD repeat-containing protein 1 (Fragment) OS=Homo sapiens GN=GRWD1 PE=1 SV=1 | 53 | 25599 | 2 | 2 | 9,2 | 4,76 |
| P07900 | Heat shock protein HSP 90-alpha OS=Homo sapiens GN=HSP90AA1 PE=1 SV=5 | 50 | 84929 | 2 | 2 | 3,3 | 4,94 |
| A0A0A0MT26 | Sodium/potassium-transporting ATPase subunit alpha-3 OS=Homo sapiens GN=ATP1A3 PE=1 SV=1 | 49 | 134795 | 1 | 1 | 1,2 | 5,76 |
| P82663 | 28S ribosomal protein S25, mitochondrial OS=Homo sapiens GN=MRPS25 PE=1 SV=1 | 49 | 20287 | 1 | 1 | 6,4 | 8,99 |
| B4DUR8 | T-complex protein 1 subunit gamma OS=Homo sapiens GN=CCT3 PE=1 SV=1 | 48 | 56053 | 1 | 1 | 3,8 | 5,47 |
| P82673 | 28S ribosomal protein S35, mitochondrial OS=Homo sapiens GN=MRPS35 PE=1 SV=1 | 48 | 37051 | 1 | 1 | 3,1 | 8,4 |
| Q92665 | 28S ribosomal protein S31, mitochondrial OS=Homo sapiens GN=MRPS31 PE=1 SV=3 | 48 | 45382 | 1 | 1 | 5,8 | 9,32 |
| A0A0A0MRA5 | Heterogeneous nuclear ribonucleoprotein U-like protein 1 OS=Homo sapiens GN=HNRNPUL1 PE=1 SV=1 | 47 | 86346 | 1 | 1 | 1,8 | 8,94 |
| P60059 | Protein transport protein Sec61 subunit gamma OS=Homo sapiens GN=SEC61G PE=1 SV=1 | 47 | 7782 | 1 | 1 | 17,6 | 10,01 |
| O14896 | Interferon regulatory factor 6 OS=Homo sapiens GN=IRF6 PE=1 SV=1 | 46 | 53555 | 1 | 1 | 4,1 | 5,18 |
| Q9BRT6 | Protein LLP homolog OS=Homo sapiens GN=LLPH PE=1 SV=1 | 44 | 15307 | 1 | 1 | 9,3 | 10,38 |
| E7ESE2 | Prohibitin (Fragment) OS=Homo sapiens GN=PHB PE=1 SV=2 | 43 | 22431 | 1 | 1 | 4,5 | 5,97 |
| Q9P0K7 | Ankycorbin OS=Homo sapiens GN=RAI14 PE=1 SV=2 | 43 | 110479 | 1 | 1 | 2,3 | 5,87 |
| H0YCK3 | Double-stranded RNA-specific adenosine deaminase (Fragment) OS=Homo sapiens GN=ADAR PE=1 SV=1 | 42 | 133530 | 1 | 1 | 1 | 8,71 |
| P60468 | Protein transport protein Sec61 subunit beta OS=Homo sapiens GN=SEC61B PE=1 SV=2 | 42 | 10014 | 1 | 1 | 15,6 | 11,57 |
| I3L3B0 | Complement component 1 Q subcomponent-binding protein, mitochondrial OS=Homo sapiens GN=C1QBP PE=1 SV=1 | 41 | 20168 | 1 | 1 | 7,3 | 4,1 |
| U3KQF2 | ADP-ribosylation factor 4 OS=Homo sapiens GN=ARF4 PE=1 SV=1 | 41 | 8072 | 1 | 1 | 11,3 | 4,81 |
| Q96BK5 | PIN2/TERF1-interacting telomerase inhibitor 1 OS=Homo sapiens GN=PINX1 PE=1 SV=2 | 40 | 37150 | 1 | 1 | 4,9 | 9,61 |
