# Supplementary material for: IFN alpha inducible protein 27 (IFI27) acts as a positive regulator of PACT-dependent PKR activation after RNA virus infections
Source: PLoS Pathog. 2025 Jun 16;21(6):e1013246. doi: 10.1371/journal.ppat.1013246 (PMC12204625; doi:10.1371/journal.ppat.1013246)
Supplement: Supplementary file 3 [file ppat.1013246.s008.docx]

Category	Term	Count	%	PValue	Fold Enrichment	FDR	Genes
GOTERM_BP_DIRECT	GO:0007015~actin filament organization	11	13.253012048192772	4,49E-10	18,02329114	2,69E-07	O94832, A0A0A0MRM8, P68032, Q16643, H0YKX5, P67936, Q9ULJ8, E9PDF6
GOTERM_BP_DIRECT	GO:0030048~actin filament-based movement	6	7.228915662650602	1,36E-08	73,73164557	4,06E-06	Q7Z406, E9PDF6
GOTERM_BP_DIRECT	GO:0030050~vesicle transport along actin filament	5	6.024096385542169	1,68E-07	94,52775073	3,34E-05	E9PDF6
GOTERM_BP_DIRECT	GO:0006412~translation	9	10.843373493975903	2,74E-06	10,10022542	4,10E-04	P19525, P32969, A0A087WVQ9, A0A087WXM6, P62241, M0R1A7, X6R631, P62280, I3L3P7
GOTERM_BP_DIRECT	GO:0030049~muscle filament sliding	4	4.819277108433735	1,34E-05	81,92405063	0,00144351	H0YKX5
GOTERM_BP_DIRECT	GO:0006936~muscle contraction	6	7.228915662650602	1,45E-05	19,15107677	0,00144351	P14649, H0YKX5, P67936
GOTERM_BP_DIRECT	GO:0002181~cytoplasmic translation	6	7.228915662650602	3,44E-05	16,0286186	0,00285207	P62280, I3L3P7
GOTERM_BP_DIRECT	GO:0032543~mitochondrial translation	6	7.228915662650602	3,82E-05	15,68758416	0,00285207	V9GYA7
GOTERM_BP_DIRECT	GO:0001649~osteoblast differentiation	6	7.228915662650602	2,27E-04	10,76374388	0,01508618	P67936, H0Y6E7
