# Supplementary material for: IFN alpha inducible protein 27 (IFI27) acts as a positive regulator of PACT-dependent PKR activation after RNA virus infections
Source: PLoS Pathog. 2025 Jun 16;21(6):e1013246. doi: 10.1371/journal.ppat.1013246 (PMC12204625; doi:10.1371/journal.ppat.1013246)
Supplement: Supplementary file 4 [file ppat.1013246.s009.docx]

Category	Term	Count	%	PValue	Fold Enrichment	FDR	Genes
GOTERM_MF_DIRECT	GO:0003723~RNA binding	42	50.602409638554214	1,06E-24	6,679052208	2,72E-22	Q96SI9, Q9BRT6, Q15633, B7Z645, P07900, P32969, A0A1B0GTT5, O75569, P62280, Q9Y2Q9, O95793, P19525, H0YKS4, I3L1L3, A0A087WVQ9, A0A087WXM6, M0R1A7, F8VR82, D6RJ07, I3L3P7, P27824, A0A1X7SBS1, Q15366, H0Y6E7, P56182, F5GXS2, O43143, A0A0D9SF53, A0A0A0MRA5, J3KQE5, M0QX71, O75475, Q5T6W2,
GOTERM_MF_DIRECT	GO:0051015~actin filament binding	17	20.481927710843372	1,21E-15	17,69842818	1,55E-13	F5GXS2, O15145, O94832, B1AK87, Q6WCQ1, Q9UHB6, Q9ULJ8, Q7Z406, A0A087WWU8, H0YK48, F5H6E2, P52907, A0A0A0MRM8, Q16643, H0YKX5, P67936, F8WCF6, E9PDF6
GOTERM_MF_DIRECT	GO:0003779~actin binding	18	21.686746987951807	3,98E-14	12,58623953	3,41E-12	Q9NYL9, F5GXS2, O15145, O94832, B1AK87, Q7Z406, A0A087WWU8, H0YK48, Q9P0K7, F5H6E2, P52907, A0A0A0MRM8, A0A1X7SBS1, Q16643, H0YKX5, P67936, F8WCF6, E9PDF6
GOTERM_MF_DIRECT	GO:0003725~double-stranded RNA binding	9	10.843373493975903	7,56E-10	28,87938523	4,86E-08	P19525, Q96SI9, O43143, Q15633, A0A1B0GTT5, O75569, D6RJ07, A0A1X7SBS1, O95793
GOTERM_MF_DIRECT	GO:0003735~structural constituent of ribosome	11	13.253012048192772	6,53E-09	13,70576025	3,36E-07	P32969, A0A087WXM6, P62241, M0R1A7, Q92665, P82673, P82663, X6R631, P62280, I3L3P7, V9GYA7
GOTERM_MF_DIRECT	GO:0000146~microfilament motor activity	7	8.433734939759036	1,15E-08	43,15019255	4,95E-07	G8JLA2, F5H6E2, O94832, A0A0A0MRM8, P68032, Q7Z406, E9PDF6
GOTERM_MF_DIRECT	GO:0003774~cytoskeletal motor activity	7	8.433734939759036	3,33E-08	36,43794038	1,22E-06	G8JLA2, F5H6E2, P14649, O94832, A0A0A0MRM8, Q7Z406, E9PDF6
GOTERM_MF_DIRECT	GO:0045296~cadherin binding	10	12.048192771084338	8,25E-06	7,297317833	2,65E-04	A0A0D9SF53, P11142, P52907, J3KQE5, B1AK87, Q5T6W2, Q16643, Q6WCQ1, Q9UHB6, E9PDF6
GOTERM_MF_DIRECT	GO:0008307~structural constituent of muscle	5	6.024096385542169	3,20E-05	27,23766307	8,47E-04	A0A087WWU8, H0YK48, G8JLA2, P14649, H0YKX5, P67936
GOTERM_MF_DIRECT	GO:0005515~protein binding	74	89.1566265060241	3,30E-05	1,268035756	8,47E-04	Q9NYL9, B7Z645, P07900, A0A0C4DGB6, A0A087X035, P62280, Q6WCQ1, Q9UHB6, O95793, Q9ULJ8, A0A087WZR1, P19525, H0YKS4, Q86SG5, A0A087WVQ9, A0A087WXM6, D6RJ07, P60059, P27824, I3L3P7, H0Y6E7, A0A0A0MT26, F8WCF6, A0A0D9SF53, M0QZ52, P14649, J3KQE5, O94832, B1AK87, P68032, E7ESE2, V9GYA7, P11142, Q9P0K7, B4DUR8, P62241, P82673, Q16643, P67936, P60468, E9PDF6, Q96SI9, Q9BRT6, Q15633, G8JLA2, P32969, A0A1B0GTT5, O15145, Q5SSJ5, O75569, C9JI87, A0A087WWU8, H0YK48, F5H6E2, M0R1A7, A0A0A0MRM8, F8VR82, A0A1X7SBS1, I3L3B0, Q15366, P56182, F5GXS2, O43143, H3BTH6, A0A0A0MRA5, P40939, M0QX71, O75475, Q96BK5, Q5T6W2, U3KQF2, E9PFP8, P52907, Q92665, O14896, H0YKX5
GOTERM_MF_DIRECT	GO:0003729~mRNA binding	8	9.63855421686747	1,14E-04	7,20750469	0,00265872	A0A0D9SF53, P07900, E9PFP8, Q5T6W2, I3L3B0, H0Y6E7, Q15366, O95793
GOTERM_MF_DIRECT	GO:0019904~protein domain specific binding	7	8.433734939759036	3,19E-04	7,521593198	0,00682722	A0A1B0GTT5, O94832, J3KQE5, Q92665, F8VR82, Q5T6W2, H0Y6E7
GOTERM_MF_DIRECT	GO:0070883~pre-miRNA binding	3	3.614457831325301	6,20E-04	78,08130081	0,01226268	Q15633, O75569, J3KQE5
GOTERM_MF_DIRECT	GO:0019899~enzyme binding	8	9.63855421686747	0,00101337	5,010564758	0,01860266	P11142, Q15633, A0A0A0MRA5, O75569, D6RJ07, E7ESE2, Q15366, F8WCF6
GOTERM_MF_DIRECT	GO:0005200~structural constituent of cytoskeleton	5	6.024096385542169	0,00120713	10,64745011	0,02068218	A0A087WWU8, H0YK48, A0A1B0GTT5, O15145, H0YKX5, F8WCF6
