# Supplementary material for: IFN alpha inducible protein 27 (IFI27) acts as a positive regulator of PACT-dependent PKR activation after RNA virus infections
Source: PLoS Pathog. 2025 Jun 16;21(6):e1013246. doi: 10.1371/journal.ppat.1013246 (PMC12204625; doi:10.1371/journal.ppat.1013246)
Supplement: Attachment — Submitted filename: Reviewers response_21MAR25-MLD.pdf [file ppat.1013246.s011.pdf]

Dr Marta L. DeDiego,
Department of Molecular and Cellular Biology
Centro Nacional de Biotecnología - CSIC,
Darwin 3, Campus de Cantoblanco,
Madrid E-28049, Spain.

Tel (+34) 91-585-4552
Email marta.lopez@cnb.csic.es

Drs. Peter Sarnow (Academic Editor), Alexander Gorbalenya (Section Editor), Sumita Bhaduri-McIntosh (Editor-in-Chief), Michael Malim (Editor-in-Chief), **Plos Pathogens**

March 21st, 2025
Manuscript ID number: PPATHOGENS-D-24-02606

Dear Editors,

Please find attached the revised version of the manuscript formely titled "**IFN alpha inducible protein 27 (IFI27) acts as a positive regulator of PACT-dependent PKR activation**" and now entitled "**IFN alpha inducible protein 27 (IFI27) acts as a positive regulator of PACT-dependent PKR activation after RNA virus infections**" by López-García *et al.*, which we would like to resubmit to be further considered for publication at **Plos Pathogens.**

We would like to thank the referees and the editors for their helpful comments, and for giving us the opportunity to revise our paper. To address the issues raised by the reviewers, we have performed additional experiments, modified the figures and made changes to the text where appropriate. Attached you will find a complete point-by-point reply to reviewers' comments.

We think that the manuscript has been significantly improved and we have included several new experimental data. Therefore, we hope that in the light of the changes made, you will now find our manuscript suitable for publication at **Plos Pathogens**.

Looking forward to hearing from you soon, sincerely,

Marta L. DeDiego

marta.lopez@cnb.csic.es

Campus Universidad Autónoma de Madrid
Darwin 3, Cantoblanco, 28049 Madrid, Spain.
Tel.: +34 91 5855307

Editor Comments:

1. Please consider acknowledging RNA virus infection(s) in the title.
Response: according to the editor´s comment, we have changed our title to: IFN alpha inducible protein 27 (IFI27) acts as a positive regulator of PACT-dependent PKR activation **after RNA virus infections.**

Point-by-point reply to the reviewers' comments

Part I - Summary

Reviewer #1

General comments:
Reviewer #1: The authors have investigated the role of IFI27 in PKR mediated stress responses including translational control and stress granule formation. They have shown that IFI27 interacts with PKR and PACT using high throughput mass spectrometry and co-immunoprecipitation experiments. They demonstrate that overexpression of IFI27 can promote PKR activity and inhibit cellular translation and stress granule formation while depletion of IFI27 inhibits PKR activity and stimulates cellular translation and stress granule formation. They further show that PACT is important in mediating these responses using double knockouts. However, the effects are marginal in several places and the quantification plots do not reflect what is seen in the western blot/ microscopy images. Importantly, it is unclear why IFI27 is able to modulate translation even in the absence of poly I:C stimulation or PKR activation given that IFI27 can only interact with PACT in a RNA-dependent manner.
Response: The reviewer´s concerns are addressed as indicated below

Reviewer #2

General comments:
Reviewer #2: The manuscript by Lopez-Garcia et al. describes the identification of IFI27 as a putative novel interacting protein for dsRNA-dependent PACT-mediated PKR activation. The primary identification of IFI27-specific protein-protein interactions (PPI) were based on overexpressed HA-tagged IFI27 in HEK 293T cells followed by mass spectrometry. Subsequently, the authors performed multiple gain- and loss-of protein function experiments in presence and absence of viral or synthetic RNA molecules to provide evidence for IFI27 functions. The manuscript is overall well written with some minor language issues. Although the identification of IFI27 is interesting and relevant, I find the manuscript quite preliminary. The impact of the finding is limited due to the application of transfection methods in all experiments. In addition, the authors did not consider dose- and time-dependent effects which I find crucial when investigating an apoptosis-inducing signaling pathway. In would also recommend to perform a mutagenesis study to define the IFI27 interacting domains to provide impactful and a complete data set.
Response: we thank this reviewer by indicating that "The manuscript is overall well written with some minor language issues", and that "the identification of IFI27 is interesting and relevant". The reviewer´s concerns are addressed as indicated below.

Reviewer #3

General Comments:

marta.lopez@cnb.csic.es

Campus Universidad Autónoma de Madrid
Darwin 3, Cantoblanco, 28049 Madrid, Spain.
Tel.: +34 91 5855307

Reviewer #3: The study titled "IFN alpha inducible protein 27 (IFI27) acts as a positive regulator of PACT-dependent PKR activation" explores a novel mechanism in the regulation of the antiviral protein kinase R (PKR), which is activated during viral infections. PKR plays a critical role in the innate immune response by inhibiting protein translation upon activation, impairing viral replication, and modulating host immunity.
This manuscript offers significant contributions to the understanding of PKR regulation in antiviral defense. The work presents a compelling and innovative exploration of the role of IFI27 as a positive regulator of PACT-dependent PKR activation, significantly advancing our understanding of antiviral immunity.
This work not only highlights a novel mechanism of PKR activation but also opens avenues for potential therapeutic interventions targeting RNA-virus infections. The clarity and depth of the research make it a valuable contribution to the field of innate immunity and virology.
Response: We thank the reviewer by pointing out that "This manuscript offers significant contributions to the understanding of PKR regulation in antiviral defense. The work presents a compelling and innovative exploration of the role of IFI27 as a positive regulator of PACT-dependent PKR activation, significantly advancing our understanding of antiviral immunity. This work not only highlights a novel mechanism of PKR activation but also opens avenues for potential therapeutic interventions targeting RNA-virus infections. The clarity and depth of the research make it a valuable contribution to the field of innate immunity and virology".

Part II- Major Issues: Key Experiments Required for Acceptance

Reviewer #1

Specific comments:

1. Shorten introduction to clearly define hypothesis- The rationale behind the study is not clear- Do the authors want to study IFI27 functions in general or only with respect to its ability to modulate PKR and PACT? Why do you hypothesize that IFI27 will modulate PKR/PACT functions in the first place?

Response: As suggested by the reviewer, we have clarified in the last paragraph of the introduction that "In this manuscript, we aim to define IFI27 functions, starting from a proteomics analysis of IFI27-binding proteins". In addition, we have clarified in the first results section that: "In order to identify possible functions of IFI27 within the innate immune responses against viral infections, we performed mass spectrometry analysis of IFI27-binding proteins, and all the proteins with a score higher than 40 were analysed by Gene Ontology, using NIH DAVID software (https://davidbioinformatics.nih.gov/). Proteins were classified by biological functions (Supplementary Table 2) and molecular functions (Supplementary Table 3), and we selected all those groups of proteins whose function showed a False Discovery Rate (FDR) lower than 0.05. RNA binding (GO:0003723~RNA binding) was the main molecular function identified within the IFI27 interactome, representing 50.6% of all the proteins found, and showing an FDR of 2.27E-22 (Supplementary Table 3). This was remarkable, as we have recently described that IFI27 is a novel dsRNA binding protein (PMID: 37187533). RNA binding is closely related to virus detection and translational processes within cells (PMID: 30673536; PMID: 35381329; PMID: 40050049), so it was very interesting to find that one of the most overrepresented biological functions within IFI27 interactome was translation (GO:0006412, accounting for 10.8% of found proteins, and with a FDR of 4.1E-4) (Supplementary Table 2). Among all the proteins that were part of both terms (Translation and RNA binding, Supplementary Tables 2 and 3, respectively), we were particularly interested in analysing the presence of two of them: PKR and PACT

marta.lopez@cnb.csic.es

Campus Universidad Autónoma de Madrid
Darwin 3, Cantoblanco, 28049 Madrid, Spain.
Tel.: +34 91 5855307

endogenous proteins, as these two proteins showed very high scores (Supplementary Table 1 and Fig. 1A).

2. Fig 1A: Complete mass spec data should be made available as a supplementary table so that the ranking of PKR and PACT can be seen. The authors don't comment on any of the other proteins involved in PKR/PACT mediated pathwyas or translation control pathways that might have appeared in the mass spec results and could be regulated by IFI27.

Response: We agree with this reviewer comment. Accordingly, we have introduced a supplemental table (Supplementary Table 1) with all the proteins identified as IFI27-binding partners in the mass spectrometry analysis. In addition, all these proteins with a score higher than 40 were analysed by Gene Ontology, using NIH DAVID software (https://davidbioinformatics.nih.gov/). Proteins were classified by biological functions (Supplementary Table 2) and molecular functions (Supplementary Table 3), and we selected all those groups of proteins whose function showed a False Discovery Rate (FDR) lower than 0.05. RNA binding (GO:0003723~RNA binding) was the main molecular function identified within the IFI27 interactome, representing 50.6% of all the proteins found, and showing an FDR of 2.27E-22 (Supplementary Table 3). This was remarkable, as we have recently described that IFI27 is a novel dsRNA binding protein (PMID: 37187533). RNA binding is closely related to virus detection and translational processes within cells (PMID: 30673536; PMID: 35381329; PMID: 40050049), so it was very interesting to find that one of the most overrepresented biological functions within IFI27 interactome was translation (GO:0006412, accounting for 10.8% of found proteins, and with a FDR of 4.1E-4) (Supplementary Table 2). Among all the proteins that were part of both terms (Translation and RNA binding, Supplementary Tables 2 and 3, respectively), we were particularly interested in analysing the presence of two of them: PKR and PACT endogenous proteins, as these two proteins showed very high scores of 565, and 494, respectively (Supplementary Table 1 and Fig. 1A). As control, PKR and PACT were not detected after immunoprecipitating IFI44-HA in the cells overexpressing IFI44-HA (data not shown), strongly suggesting that the binding of PACT to IFI27 is not mediated by the HA tag. Exponentially modified Protein Abundance Index (emPAI), which estimates the absolute protein amount by analyzing the number of observed peptides divided by the number of observable peptides per protein (Ishihama et al., 2005), was higher for PACT (emPAI 1,92), than for PKR (emPAI 0,71) (Fig. 1A), suggesting that the abundance of PACT in the sample was higher than PKR abundance. Interestingly, the protein phosphatase 1C (PPP1CC), encoding the gamma isoform of the phosphatase catalytic subunit, was identified as well, although to lower protein score (279) (Supplementary Table 1). PPP1CC dephosphorylates eIF2α (PMID: 9023344). Also, TARBP2 Subunit of RISC Loading Complex (TARBP2) was found within the interactome with a protein score of 373. This protein is known to interact with PACT and modulate PKR activity (PMID: 30390472). Furthermore, we found some ribosomal proteins, which more likely are involved in protein translation (PMID: 20094052, PMID: 33383105). However, because the lower score for these proteins, in this study we decided to first focus on the likely effect of IFI27 on PKR and PACT functions.

3. Fig1B: Why was endogenous IFI27 not pulled down after poly I:C stimulation?

Response: We have performed new experiments and tried to pull-down endogenous IFI27, but unfortunately, after immunoprecipitating PACT, we have not been able to detect IFI27 by mass spectrometry. We think this could be because IFI27 is expressed to low levels in the cells we are using, and also because this protein is an small protein (12 kDa), which is more difficult to detect

marta.lopez@cnb.csic.es

Campus Universidad Autónoma de Madrid
Darwin 3, Cantoblanco, 28049 Madrid, Spain.
Tel.: +34 91 5855307

by trypsin digestion and mass spectrometry than other bigger proteins. Nevertheless, to further confirm an interaction of IFI27 with PACT in different conditions, we have performed new experiments in which we have confirmed the co-immunoprecipitation of PACT-FLAG and IFI27-HA in SARS-CoV-2-infected cells and using a FLAG antibody for the immunoprecipitation (Supplementary Fig. 1), instead of the HA antibody we used in previous co-immunoprecipitations, further supporting an interaction of IFI27 with PACT.

4. Fig 1C: Is the interaction between IFI27 and PKR RNA-dependent?

Response: Please note, that the interaction of PKR with IFI27 is mediated by PACT (Fig. 9), and that the interaction of IFI27 with PACT is RNA-dependent (Fig. 1B, and new Figures 1C and 1D). Therefore, it is expected that the interaction of IFI27 with PKR is RNA-dependent. To answer this question, we have performed new co-immunoprecipitation assays using a PKR antibody and cellular extracts originated from mock-transfected cells and from cells transfected with poly(I:C). The results show that the interaction of IFI27 with PKR is increased in the presence of poly(I:C) (Fig. 1E), an analog of dsRNA, strongly suggesting that the interaction of IFI27 with PKR is facilitated by RNAs. This new information has been introduced in the new Figure 1E, and in the corresponding materials, and results sections.

5. Fig 2A: Add a schematic of the luciferase constructs and the experimental pipeline above the graph for better understanding.

Response: Following the reviewer recommendation, we have introduced a schematic image of the plasmid constructs and the pipeline for the experiment in the revised Fig. 2A, corresponding to Fig. 3A in the revised version.

6. Fig 2B:
a. Why are mock and SARS-infected lysates run on separate gels? This doesn't allow us to compare levels of P-eIF2-alpha, P-PKR and PACT between mock and infected cells. Since the blots are separate, band intensity quantification can't be combined into one.
b. What happens to endogenous IFI27 levels upon infection?
c. How is IFI27 overexpression able to increase levels of P-PKR and P-eIF2-alpha even in the absence of dsRNA in mock cells, given that its interactions with PACT is RNA dependent?

Response: (a) According to the reviewer comment, we have run the samples in Fig. 2B in the same gel, and have provided a new gel for Fig. 2B (new Fig. 3C in the revised version), and a new quantification by densitrometry.

(b) Endogenous IFI27 levels are increased after SARS-CoV-2 infection. This information has been added to the results section, as it was previously shown, in our previous publication, that endogenous levels of IFI27 are increased at 24, and 48 hpi (PMID:37187533).

(c) Please notice that, although the interaction of IFI27 with PACT and with PKR is stronger in poly(I:C)-transfected cells (Figs. 1B and 1E, respectively), we can detect an RNA-mediated interaction of IFI27 with PACT and of IFI27 with PKR even in mock-infected cells (Figs. 1B and 1E, respectively), suggesting that endogenous RNAs may be mediating this interaction, and therefore, PKR activation, although to a weaker level. Supporting this hypothesis, it has been published a non-canonical PKR activation by endogenous RNAs, such as nuclear and

marta.lopez@cnb.csic.es

Campus Universidad Autónoma de Madrid
Darwin 3, Cantoblanco, 28049 Madrid, Spain.
Tel.: +34 91 5855307

mitochondrial RNAs (PMID: 30174290; PMID: 24939934), and microRNAs (PMID: 24554719). Furthermore, it has been very recently published in a preprint that PACT is a direct and specific suppressor of PKR against endogenous dsRNA ligands, which activate PKR in the absence of PACT (https://www.biorxiv.org/content/10.1101/2024.10.23.619951v1).

7. Fig2D:
a. Why is IFI27 expression so much higher in VSV infected cells at 8hpi compared to 24hpi?
b. Mock- infected cells should be included in the blot.
c. The changes in the blot appear to be very marginal and densitometry doesn't reflect this. Error bars in the densitometry graphs are also not clear. Western blots and densitometries need to be repeated.

Response: (a) It has been extensively described that VSV infection induces cytotoxycity and cellular shut off (for example, PMID: 15629720, PMID: 11104816, PMID: 11046154, PMID: 11106761). Therefore, the higher expression of IFI27 at 8 hpi, compared to 24 hpi, can be attributed to the latter time of infection and the induction of shut off at later times. However, we have now performed infections at a lower MOI (0.1 in the revised version, instead of 3 in the former version), and the differences in expression after 8 hpi compared to 24 hpi are produced to a much lower extent, likely attribuable to the decreased cytotoxicity and cellular shut off produced at a lower MOI (Fig. 3F in the revised version).

(b and c) According to this reviewer comment, mock-infected cells have been included in the blot and Western blots and densitometris have been repeated in figure 2D (Fig. 3F in the revised version).

8. Fig 3B: Does expression of IFI27 in KO cells rescue phenotype?

Response: We concur with the reviewer that the answer to this question is important and would reinforce our results. According to this comment, we have performed an additional experiment in which we have used IFI27 KO cells stably transfected with an empty plasmid, and IFI27 KO cells stably expressing IFI27-HA by means of a linearized plasmid transfection, previously generated and described by us (PMID: 39431052). We have transfected these cells with the plasmid expressing the Renilla luciferase, and then, the cells were treated with different concentrations of poly(I:C). The results show that, as expected given our previous results, the overexpression of IFI27 in the IFI27 KO cells decreases renilla luciferase expression, rescuing the phenotype. These new results have been introduced in the results section, and in the new Figure 4C.

9. Fig 3-4:
a. Western for IFI27 is missing in KO/KD experiments to test if the protein is depleted.
b. Why is luciferase activity increasing in mock cells upon IFI27 KD when there is no change in P- PKR or P-eIF2-alpha levels. This is also contradictory to what was seen in IFI27 overexpressing mock cells in Fig 2B.
c. Figures 3 and 4 can be combined into one.

Response: (a) Although we agree with this reviewer, we prefer not to show again published data, therefore, we have introduced in the results section a sentence to indicate that "We confirmed that the IFI27 KO cells did not expressed the IFI27 protein by Western blot (PMID:37187533)".

marta.lopez@cnb.csic.es

Campus Universidad Autónoma de Madrid
Darwin 3, Cantoblanco, 28049 Madrid, Spain.
Tel.: +34 91 5855307

(b) Please note that according to Figure 5D (Figure 4D in the former version) there existed differences in the levels of P-PKR between mock-treated cells KD for IFI27 compared to mock-treated, control cells (Figure 5D), correlating with the observed differences in Rluc expression. The fact that we observe no differences P-eIF2-alpha, could be due to the fact that these differences are small and the Western blot approach is not sensitive enough to detect them. In addition, as we have indicated in the revised version, IFI27 interacts with other proteins, including ribosomal proteins (Supplementary Table 1), which may have an additional effect of protein translation.

(c) Please note that in order to improve our manuscript, we have added new panels (panels C and D, in former Figure 3, which is now Figure 4). Therefore, because of these additions, we prefer not to combine current figures 4 and 5 (previous figures 3 and 4) in the revised version.

10. Fig 4B: Can PACT overexpression rescue the phenotype?

Response: It may be PACT overexpression rescues the phenotype. However, we have not given priority to perform this experiment. The reasons behind this decision are that we need to transfect the cells twice with the siRNAs to silence IFI27 expression, and the fact that PACT is expressed at endogenous levels in the A549 cells (Fig. 5B in the revised version), therefore, it could be that the differences at the level of PACT overexpression are not detected, giving us unconclusive results.

11. Fig 5: Why is luciferase decreasing in mock cells upon IFI27 overexpression when there is no change in P-PKR or P-eIF2-alpha?

Response: Please note that we have quantified again, more carefully using different expositions, the levels of P-PKR, showing an increased in P-PKR in the mock-treated cells overexpressing IFI27, compared to the control cells transfected with the empty plasmid (new quantification in Fig. 6B in the revised version, corresponding to former figure 5B). The fact that we observe no differences P-eIF2-alpha, could be due to the fact that these differences are small and the Western blot approach is not sensitive enough to detect them. In addition, as we have indicated in the revised version, IFI27 interacts with other proteins, including ribosomal proteins (Supplementary Table 1), which could have an additional effect of protein translation.

12. Fig 6: Once again, the microcopy images are not reflective of what is seen with quantification. Increase in G3BP1 or eIF3A is not evident in the images shown.

Response: Please, note that we don´t observe an increase in the intensity of G3BP1 or eIF3n staining, in cells overexpressing IFI27, compared to control cells. What we observe is an increase in the number of SG formation, visualized by the staining of G3BP1 and eIF3n using specific antibodies. In any case, taking into account this referee´s comment, we have changed the images in figure 6B (Figure 7B in the revised version), by introducing higher magnification images, and by selecting images in which the presence of stress granules, observed by the staining of G3BP1 and eIF3n, is higher in the WT cells overexpressing IFI27, than in the WT cells not overexpressing IFI27. Please, note that we had already counted the percentage of cells containing stress granules, by counting hundreds of different cells for each condition (currently in Fig. 7C), and the changes in the images does not affect the results previously reported.

13. Fig 6 and 7 convey the same thing and can be combined into one.

marta.lopez@cnb.csic.es

Campus Universidad Autónoma de Madrid
Darwin 3, Cantoblanco, 28049 Madrid, Spain.
Tel.: +34 91 5855307

Response: According to this reviewer´s comment, we have merged figures 6 and 7 into one (Figure 7 in the revised version).

14. Fig 8: Images need be better representative

Response: taking into account this referee´s comment, we have changed the images in figure 8C (same numbering in the revised version), by introducing higher magnification images, and by selecting images in which the presence of PKR in stress granules, is higher in the WT cells overexpressing IFI27 and PKR, than in the WT cells overexpressing PKR alone. Please, note that we had already counted the percentage of cells containing stress granules, by counting hundreds of different cells for each condition, and the changes in the images does not affect the results previously reported.

Reviewer #2

Specific Comments:
1. The initial experiment was done by transfecting HA-tagged IFI27 in HEK-293T cells which have low level MDA5. Since MDA5 can activate PACT, the very first experiment already includes an experimental bias. In addition, the authors should rule out any effect of the HA-tag by confirming the results with a different tagging and untagged versions. Please also confirm the finding in at least a second cell line, preferably primary human cells (see point 2).

Response: Please, take into consideration that in addition to HEK293T cells, the binding of IFI27 to PKR has been also shown in the Hela cells (Fig. 9). In addition, we have performed experiments in A549 and Hela cells showing that IFI27 positively affects PKR activation and protein translation (Figs. 4, 5, 6, and 8). Nevertheless, taking into account this consideration, we have acknowledged in the first paragraph of the discussion that "the experiments have been performed in established cell lines and more experiments in primary cells or even in vivo are required to provide a more comprehensive understanding". In addition, we have specified in the abstract section that we have used tumour-derived, established cells.

Furthermore, in previous mass spectrometry experiments, we included cells transfected with the empty plasmid, cells transfected with a plasmid expressing IFI44 fused to an HA tag, and cells transfected with the plasmid expressing IFI27 fused to the HA tag. Interestingly, we identified PACT in the cells overexpressing IFI27-HA, but not in the cells overexpressing IFI44-HA, strongly suggesting that the binding of PACT to IFI27 is not mediated through to the HA tag. This new information has been included in the revised version of the manuscript, in the result´s first paragraph. Furthermore, please note that we have included new experiments (Figs. 1C and 1D) in which we have analyzed the binding of two IFI27 variants to PACT. These two variants encode only one single amino acid mutation with respect to the IFI27-WT, and the same HA tag as the IFI27-WT protein. However, these variants are defective in binding to poly(I:C) and PACT, again, supporting the idea that the binding of the IFI27-WT protein to PACT is specific and is not dependent on the the HA tag.

2. Transfections stress cells◊ Due to this exogenous stress, PACT might already be phosphorylated at positions S246 and S287, which increases PKR activation (see e.g. review PMID: 33387379). Please rule out that the transfection procedures do not include a bias. I would recommend to confirm endogenous PPI analyses. I would use +/- IFN treated cells (optimally primary cells, not

marta.lopez@cnb.csic.es

Campus Universidad Autónoma de Madrid
Darwin 3, Cantoblanco, 28049 Madrid, Spain.
Tel.: +34 91 5855307

cancer cells). IP could be done using commercial PKR antibodies for pull down and perform MS afterwards. This would show PPI under more native conditions avoiding transfection procedures.

Response: Please, note that to avoid transfection bias, transfections with the same amount of an empty plasmid, and lipofectamine, as control, are included in all of our experiments. In any case, to rule out that transfection experiments do not include a bias, we have performed experiments in WT and IFI27 KO cells infected with SARS-CoV-2, without any transfection, showing that the absence of IFI27 decreases the levels of phospho-PKR after SARS-CoV-2 infection (new Fig. 4D). In addition, please note that we have used IFI27 KO cells (Figs. 4A, and 4B), and cells stably expressing IFI27 (new Fig. 4C). In these cells we do not transfect plasmids, and show that IFI27 positively affects PKR activation. Furthermore, our results show that after transfecting the IFI27 plasmid, the levels of luciferase are reduced in Hela WT cells, but they are reduced to much lower effects or to no effects, in Hela PACT KO and Hela PKR KO cells (Fig. 6A). In addition, after IFI27 overexpression and poly(I:C)-transfection, the levels of phospho-PKR and phospho-eIF2α are increased in the Hela WT cells, whereas they remain unchanged in the Hela PACT KO cells (Fig. 6B), strongly suggesting that the effect of IFI27 overexpression on PKR activation, even in transfected cells, is specific and highly depends on PACT expression.

In addition, we have tried to pull-down endogenous IFI27, but unfortunately, after immunoprecipitating PACT, we have not been able to detect it by mass spectrometry. We think this could be because IFI27 is expressed to low levels in the cells we are using, and also because this protein is an small protein (11 kDa), which is more difficult to detect by trypsin digestion and mass spectrometry than other bigger proteins. Nevertheless, to further confirm an interaction of IFI27 with PACT in different conditions, we have performed new experiments in which we have confirmed the co-immunoprecipitation of PACT-FLAG and IFI27-HA in SARS-CoV-2-infected cells and using a FLAG antibody for the immunoprecipitation (new Supplementary Fig. 1), instead of the HA antibody we used in previous co-immunoprecipitations, further supporting an interaction of IFI27 with PACT.

3. Most presented data is based on single plasmid/virus/RNA concentrations and single times points. Since PACT/PKR activation causes translational shut off, the actual transfection procedures, the plasmid concentrations, and late time points might cause apoptosis. Please include time and dose-dependent experiments for some of the crucial findings. You might also perform cell viability assays to rule out apoptosis.

Response: According to this reviewer comment, we have performed experiments at different time points and analyzed PKR phosphorylation: (i) in cells overexpressing IFI27 and infected with SARS-CoV-2 (new Fig. 3C), (ii) in cells overexpressing IFI27 and infected with VSV (new Fig. 3F), and (iii) in WT and IFI27 KO cells infected with SARS-CoV-2 (new Fig. 4D).

In addition, the effect of plasmid concentration on translational shut off has been analyzed in a new experiment by transfecting 3 different concentrations of plasmids (new Fig. S3). The results show that the higher the concentration of plasmid is, the higher the expression of IFI27 is, as expected, and the lower the levels of Renilla luciferase expression are. In addition, for a given plasmid concentration, the expression of Renilla luciferase is lower in the cells transfected with the plasmid expressing IFI27, compared to the cells transfected with the empty plasmid (new Fig. S3), as expected, given our results indicating that IFI27 positively affects PKR activation.

marta.lopez@cnb.csic.es

Campus Universidad Autónoma de Madrid
Darwin 3, Cantoblanco, 28049 Madrid, Spain.
Tel.: +34 91 5855307

4. As model viruses, the authors use SARS-CoV-2 and VSV both triggering RIG-I and MDA-5 to certain levels. I would be recommended to use viruses (e.g EMCV for MDA-5) or exogenous stimuli that exclusively trigger MDA-5 or RIG-I to evaluate the effects of RIG-I and MDA5-dependent PACT activations. This is especially important as HEK293T have low level MDA5 expression (see comment above). I would also recommend to perform Western blots for RIG-I and MDA5 to monitor proteins that influence downstream PACT activation. Response: Please, take into consideration that in addition to HEK293T cells, the binding of IFI27 to PKR has been also shown in the Hela cells (Fig. 9). In addition, we have performed experiments in other cell lines different from HEK-293T cells, such as A549 and Hela cells showing that IFI27 positively affects PKR activation and protein translation (Figs. 4, 5, 6, 7, and 8).

Furthermore, in this paper we have focused on the effect of IFI27 on PKR activation. Please note, that we have previously shown that IFI27 negativey modulate the activation of RIG-I and MDA-5 (PMID: 39431052 and PMID: 37187533). Therefore, the binding of IFI27 to PACT and to PKR (this last in a PACT-dependent manner) could lead to lower levels of free PACT able to bind to RIG-I and MDA-5, and therefore, to a lower activation of RIG-I and MDA-5, as we have previously published. However, although we think that the effect of IFI27 on PACT-mediated RIG-I and MDA-5 activation is an interesting point, we prefer to leave these experiments for further studies, as this point is not the focus of this manuscript.

5. Figure 2/Page 13: as the authors mention, SARS-CoV-2-mediated PKR activation has been well defined already. For confirmation and specification of the findings, I would strongly recommend to use a virus pair harboring defined mutations influencing PKR interactions e.g. SARS-CoV-2 N or nsp15 mutants or FluA NS1 mutants etc. In addition, at least 2 time points would be necessary to observe the dynamics of the virus-mediated interferences. This is especially important as the authors use transfection-based assays that strongly influence the interferon and PKR-dependent pathways.

Response: Please, note that we have performed new experiments including two time points after SARS-CoV-2 (Fig. 3C and 4D) and VSV (Fig. 3F) infections. In addition, we are aware that plasmid transfections affect the interferon and PKR-dependent pathways. However, and to avoid transfection bias, transfections with the same amount of an empty plasmid, and lipofectamine, as control, are included in all of our experiments. Furthermore, to analyze the effect of IFI27 on PKR activation in the absence of transfection, we have analyzed PKR phosphorylation in SARS-CoV-2 -infected cells, at two time points, in WT and IFI27 KO cells (new Figure 4D). The results show higher levels of PKR phosphorylation in WT cells, compared to IFI27 KO cells, further indicating that IFI27 positively affects PKR activation after RNA virus infections, even in the absence of cell transfections. In addition, we have similar results using VSV infections (Fig. 3F), and in the absence of infections, using poly(I:C) as an analog of dsRNAs produced during the infections by many viruses (Figs. 4B, 5D, 6B, 8D). Therefore, although we concur with the reviewer that using SARS-CoV-2 N or nsp15 mutants or FluA NS1 mutants would be interesting, we think that the results presented in the revised version further confirm that IFI27 affects the protein translation mediated by PKR, even after the infection with viruses encoding proteins inhibiting PKR activation.

6. For an impactful publications, I would expect a mutagenesis study showing how exactly IFI27 interacts (at least domain level).

marta.lopez@cnb.csic.es

Campus Universidad Autónoma de Madrid
Darwin 3, Cantoblanco, 28049 Madrid, Spain.
Tel.: +34 91 5855307

Response: to answer this concern, we have performed completely novel experiments using two IFI27 variants. According to bioinformatic predictions using RNAbindRPlus, IFI27 contains 13 amino acids which are predicted to bind RNA, which are the amino acids 60-65, 69, 82-86. To further confirm that IFI27 binds PACT in an RNA-dependent manner, we generated two IFI27 variants encoding point mutations in two of these amino acids predicted to bind RNAs (i.e. variants S63L and V82A) (new Fig. 1C and 1D). We found that whereas these two IFI27 mutants are expressed to similar levels to the WT protein, they are severily impaired in binding to poly(I:C) (Fig. 1C), confirming the informatic predictions suggesting that these two amino acids are relevant for IFI27 binding to RNAs. Remarkably, we show that these two IFI27 variants defective in poly(I:C) binding did not interact with PACT (Fig. 1D), further indicating that the binding of IFI27 to PACT is mediated by the binding of IFI27 to dsRNAs.

7. Figure 8. How do the authors explain the difference in RLUC expression between WT and IFI27 KO in mock treated cells? Did the authors confirm that transfection efficiency is equal between WT and KO? To confirm the specificity of the knockout, I would also suggest re-complementing IFI27 for example by transient overexpression in the KO cells.

Response: Please take into consideration that endogenous RNAs can activate PKR in mock-infected cells. For example, it has been published a non-canonical PKR activation by endogenous RNAs, such as nuclear and mitochondrial RNAs (PMID: 30174290; PMID: 24939934), and microRNAs (PMID: 24554719). Furthermore, it has been very recently published in a preprint that PACT is a direct and specific suppressor of PKR against endogenous dsRNA ligands, which activate PKR in the absence of PACT (https://www.biorxiv.org/content/10.1101/2024.10.23.619951v1). Nevertheless, to rule out that the differences in Rluc expression between WT and IFI27 KO cells are not due to differences in transfection efficiency between bot cell lines, we have performed an RT-qPCR experiment in which we have analyzed the expression of Rluc at the mRNA level. These new results indicate that the expression of Rluc mRNAs is the same in both cell lines, indicating that there are no significant differences in the transfection efficienty and in Rluc mRNA transcription between WT and IFI27 KO cells. These new results have been introduced in the revised version of the manuscript and in Fig. S6.

To confirm the specificity of the knockout, we have performed an additional experiment in which we have used IFI27 KO cells stably transfected with an empty plasmid, and IFI27 KO cells stably expressing IFI27-HA by means of a linearized plasmid transfection, previously generated and described by us (PMID: 39431052). We have transfected these cells with the plasmid expressing the Renilla luciferase, and then, the cells were treated with different concentrations of poly(I:C). The results show that, as expected given our previous results, the overexpression of IFI27 in the IFI27 KO cells decreases renilla luciferase expression, rescuing the phenotype. These new results have been introduced in the results section, and in new Figure 4C.

8. Figure 2D. The signal to noise ratio in 2D seems unacceptable for actin. A normalization to this is not possible. The WB should be repeated if possible. I would probably use a different reference gene/protein as Actin expression/translation is often affected by virus infections see e.g. PMID: 15705200.

Response: Taking into account this comment, and being agree with the referee, we have performed a new blot for the actin detection (new Fig. 3F, which was Fig. 2D in the former version). In

marta.lopez@cnb.csic.es

Campus Universidad Autónoma de Madrid
Darwin 3, Cantoblanco, 28049 Madrid, Spain.
Tel.: +34 91 5855307

addition, please, note that the paper mentioned by this reviewer (PMID: 15705200) show small differences at the levels of actin mRNA expression, they do not analyze this at the level of protein expression, and we find no differences in the actin expression at the protein level after VSV and SARS-CoV-2 infections (i.e. Figures 3C, 3F, and 4D), suggesting that the normalization with actin at the protein level is suitable for these assays.

9. Figure 5A and 8B: Why does the KO of PACT ("empty") lead to decreased protein translation. Shouldn't it have a similar effect as PKR KO, thereby increasing protein translation?
Response: Please, note that in Figs 5A and 8B (Figs. 6A and 8B in the revised version), we only observed decreased protein translation in the PACT KO cells, compared to the WT cells in the mock-treated cells transfected with the empty plasmid. We do not observe this effect in the poly(I:C)-treated cells transfected with the empty or IFI27 plasmids, and in the mock-treated cells transfected with the IFI27 plasmid. Although PACT is an activator of PKR under stress conditions, it has been shown in two preprints that PACT can act as a direct and specific suppressor of PKR against endogenous dsRNA ligands, which can activate PKR in the absence of PACT (PMID: 39651230 and https://doi.org/10.1101/2024.10.23.619951). Therefore, the decreased protein translation in the PACT KO, mock-treated cells transfected with the empty plasmid could be due to this PACT function of decreasing PKR activation mediated by endogenus dsRNAs or by plasmid-derived RNAs. This new information has been included in the results section of "PACT is required for the activation of PKR by IFI27 protein".

10. Figure 9. The authors show a dsRNA dependent interaction between IFI27 and PACT by including a RNAse condition in their co-IP (Figure 1). For the IFI27-PKR interaction in Figure 9 they omit the RNAse treatment and seem to claim a direct interaction. I would suggest that the authors include the RNAse treatment also in the IFI27-PKR co-IP to exclude dsRNA mediated interaction.

Response: Please, note that we don´t claim a direct interaction of IFI27 with PKR, as it seems that the reviewer suggests. We claim that the interaction of IFI27 with PKR is PACT-dependent and that the interaction of IFI27 with PACT is RNA-dependent. In any case, to answer this question, we have performed new co-immunoprecipitation assays using cellular extracts from mock-infected cells and from poly(I:C)-treated cells (new Fig. 1E). The results show that the interaction of IFI27 with PKR is enhanced in cells treated with poly(I:C) (new Fig. 1E), as we could expect, taking into account that the interaction of IFI27 with PACT is favoured in poly(I:C)-treated cells (Fig. 1B), and that the interaction of IFI27 with PKR is PACT-dependent (Fig. 9). This new information has been introduced in the new Figure 1E, and in the matherials, and results sections.

Reviewer #3

Specific comments:

1. In the immnoprecipitation experiments, the authors could analyze the interaction in the context of viral infection, not only using polyIC as a trigger.

Response: We agree with this reviewer´s comment. Accordingly, we have analyzed the interaction of IFI27 with PACT in SARS-CoV-2-infected cells. This new information, has been added to the

marta.lopez@cnb.csic.es

Campus Universidad Autónoma de Madrid
Darwin 3, Cantoblanco, 28049 Madrid, Spain.
Tel.: +34 91 5855307

results section, and in Figure S1.

2. In figure 1D, the authors should explicitly state whether the colocalization shown in Figure 1D occurs under mock-treated or poly(I:C)-treated conditions. Perform colocalization analysis both before and after poly(I:C) treatment. This would provide valuable insights into how the interaction between IFI27, PACT, and PKR changes in response to dsRNA stimulation.

Response: Please, note that the data presented in figure 1B shows that whereas the amount of PACT in the cellular extracts was similar, the amount of PACT bound to IFI27 was clearly decreased in the cells non-transfected with poly(I:C), compared to the cells transfected with poly(I:C) (Fig. 1B), indicating that poly(I:C) clearly favours the binding of IFI27 to PACT. Similarly, we have found that the binding of IFI27 to PKR is also favoured in the presence of poly(I:C) (Fig. 1E). In addition, to address this reviewer concern, we have performed new colocalization analysis of IFI27 with PACT, and of IFI27 with PKR, in mock-transfected, and in poly(I:C)-transfected cells (new Fig. 2). The colocalization area of both fluorophores (Alexa-488 for PACT-FLAG or PKR and Alexa-594 for IFI27-HA) was analyzed and divided by the total cell area (at least n=29 cells for each condition) obtaining a ratio that was transformed into percentage of colocalization. PACT-IFI27 colocalized in 8.27% of the cell area under mock conditions, a percentaje increasing to 13.6% after poly(I:C) treatment. Similarly, PKR-IFI27 colocalized in 8.38% of the cell area under mock conditions, increasing to 13.7% of colocalization after poly(I:C) treatment (Figs. 2A and 2B). These results further support the interaction of IFI27 with PACT and PKR from the MS and Co-IP experiments, and, suggest that poly(I:C) favours the interaction of IFI27 with PACT and PKR, supporting the results shown in Figures 1B and 1E.

3. Immunofluorescence and colocalization images (Figs 6 and 7 While stress granule (SG) formation is noted as a significant finding, the lack of clear co-localization of IFI27 with SG markers (e.g., G3BP1) raises questions about the direct role of IFI27 in SG formation. This requires further clarification and higher imaging quality (i.e. zoom areas or higher magnification).

Response: Please, note, that we do not think that IFI27 plays a direct role on the formation of SGs. We rather think that because IFI27 affects the activation of PKR, this effect indirectly affects SG formation, as it has been described that the translational arrest induced by activated PKR leads to the formation of SGs (Eiermann et al., 2020; Gao et al., 2022). Accordingly, we have introduced the following sentence in the revised version of the manuscript: "we determined whether IFI27 overexpression could indirectly increase the formation of SGs by positively affecting the activation of PKR".

Furthermore, taking into account this referee´s comment, we have changed the images in figure 6B (Fig. 7B in the revised version), by introducing higher magnification images, and by selecting images in which the presence of stress granules, observed by the staining of G3BP1 and eIF3n, is higher in the WT cells overexpressing IFI27, than in the WT cells not overexpressing IFI27. In this case, please, note that we had already counted the percentage of cells containing stress granules, by counting hundreds of different cells for each condition (currently in Fig. 7C), and the

marta.lopez@cnb.csic.es

Campus Universidad Autónoma de Madrid
Darwin 3, Cantoblanco, 28049 Madrid, Spain.
Tel.: +34 91 5855307

changes in the images does not affect the results previously reported.

4. The experiments focus primarily on HEK-293T, A549, and HeLa cell lines, which, while informative, may not fully capture the complexity of IFI27's role in diverse cellular or tissue-specific environments. Expanding to primary cells or animal models would provide a more comprehensive understanding.

Response: Although we concur with the reviewer comment, expanding the effect of PKR to primary and animal models is currently out of the possibilities given the time we were given to reply to the comment´s reviewers. In addition, it has to be taken into consideration that we have performed experiments in different cell lines and that, in the case of animal models, the sequence homology between human and mouse IFI27 is not so high, therefore, the IFI27 functions could not be the same in humans and mice. In any case, and taking into account this point, we have acknowledged in the first paragraph of the discussion that "the experiments have been performed in established cell lines and more experiments in primary cells or even in vivo are required to provide a more comprehensive understanding". In addition, we have specified in the abstract section that we have used tumour-derived, established cells for the experiments.

Part III – Minor Issues: Editorial and Data Presentation Modifications

Reviewer #1:

1. Quality of images and blots need to be better

Response: Following this reviewer recommendation, fluorescence images in former figures 6B and 8C (corresponding to Figures 7B and 8C in the revised version) have been improved. In addition, some of the blots (i.e. Figures 2B and 2D, corresponding to Figures 3C and 3F in the revised version) have been improved. Furthermore, we have included new blots (Fig. 4D, Fig. S3B, S3C) to further support the manuscript conclusions.

2. Writing should be more concise and articulate especially where the authors describe quantification of stress granules in IFI27 expressing PACT KO cells

Response: we have clarified in the text that "As IFI27 can potentiate PKR activation, one of the main inductors of SGs (Boccaccio et al., 2023), we determined whether IFI27 overexpression could indirectly increase the formation of SGs by positively affecting the activation of PKR". In addition, we have re-writen the paragraph describind these results, being the specific changes shown in red.

Reviewer #2:

1. Please show individual data points in all Figures. How often were experiments repeated independently?

Response: According to this reviewer comment, we have included individual data points in all the Figures related with Renilla luciferase measures, and in all the measures related to fluorescence quantification in cells. In addition, we have included in the figure legend the number the experiments were repeated.

2. Figure 6C is way too small. Figure 6 and figure 7 show the same thing with a slightly different analysis. Consider merging them. Figure 6B could be in the supplement

marta.lopez@cnb.csic.es

Campus Universidad Autónoma de Madrid
Darwin 3, Cantoblanco, 28049 Madrid, Spain.
Tel.: +34 91 5855307

Response: According to the referee recommendation we have introduced a bigger Fig. 6C in the revised version of the manuscript (currently Fig. 7C). In addition, Figures 6 and 7 have been merged (Fig. 7 in the revised version). Please, note that this recommendation was also made by reviewer 1, in his/her comment 13.

Although we respect the comment suggesting that the Fig. 6B could be in the supplement, we think that the improved Fig. 7B in the revised version of the manuscript (Former Fig. 6B) is very visual and informative, so, we prefer to leave it in the main manuscript.

3. Figure 7. How many cells were counted and how often was the experiment repeated? Please show single data points to get estimates on the distributions.
Response: The number of cells counted for each condition, using different images is presented in the revised figure 7C (formerly Figure 6C), in the x-axis of the revised figure 7E (formerly figures 7A, 7B) and in the figure 8D (formerly Figure 8D). In addition, this information has been included in the legends of Figures 7C, 7E and 8D, in the revised version.

4. Figure 8: Please explain the purpose and value of figure 8? As far as I understand it shows similar results that previous figures but just in cells that overexpress PKR. IFI27 increases the number of SGs dependent on PACT (same as fig 6) and a IFI27 KO increases protein translation (same as fig 3). In cells with artificially high PKR levels the PKR induction (and downstream translation inhibition) is slightly higher but, in my opinion, this does not add anything and is expected. It might be considered for the supplement.

Response: We think that figure 8 (same number in the revised version) reinforces the conclusions indicated from Figures 6 and 4, although in different conditions (under PKR overexpression). Because of this, and taking into account that no restriction in the number of figures apply, we prefer to leave it as it stands now.

Reviewer #3:

1. While the figures are informative, some lack detailed annotations or explanations, making interpretation more challenging for readers unfamiliar with the methods.

Response: we have introduced an schema of the plasmids and experimental design in Fig. 3A (see coment 5 from reviewer 1) so that the interpretation can be more familiar to readers. In addition, we have included additional explanations in legend figures (i.e. Figures 7C, 7E, 8D)

2. Statistical significance is noted but could benefit from expanded analysis, such as additional replicates, or indicating if they were done.

Response: According to this reviewer comment, we have included individual data points in all the Figures related with Renilla luciferase measures, and in all the measures related to fluorescence quantification in cells. In addition, we have included in the figure legend the number the experiments were repeated. In addition, we have included information about the number of cells analyzed in the corresponding figures (i.e. Figures 2B, 7C, 7E, 8D)

marta.lopez@cnb.csic.es

Campus Universidad Autónoma de Madrid
Darwin 3, Cantoblanco, 28049 Madrid, Spain.
Tel.: +34 91 5855307

3. Potential Conflict with Previous Findings: The manuscript claims that IFI27 enhances the activity of PKR, promoting antiviral responses by reducing protein translation. This contrasts with the previous findings (Villamayor et al.,2023), where IFI27 was shown to negatively regulate innate immunity, particularly by interacting with Retinoic acid-inducible gene I (RIG-I) and melanoma differentiation-associated protein 5 (MDA-5). These interactions reportedly suppress downstream immune signaling, reducing interferon (IFN) production. Reconciling these roles with additional discussion, possible explanations, or experiments would enhance the paper's coherence.

Response: PKR activation could modulate the innate immune responses after viral infections by blocking the translation of antiviral and inflammatory proteins. In this sense, the decreased PKR activation in cells knocked-in or knocked-out for IFI27 could be contributing to an increased translation of innate immune response proteins, providing, therefore, an additional molecular mechanism to the effect of IFI27 in negatively modulating innate immune responses, as we previously published (Villamayor et al., 2023; Rivero et al., 2024). This explanation has been included in the discussion section.

Although it is true that the effect of IFI27 on counteracting IFN-responses might led to decreased expression of PKR, as PKR is an IFN-induced gene and IFI27 negatively modulates the IFN responses, PKR is also constitutively expressed, and we do not detect significant differences in the levels of total PKR among cells overexpressing IFI27 (Figs. 3C, 3F, and 6B), IFI27 KO cells (Figs. 4B and 4D), IFI27 knocked-down cells (Fig. 5D), and their corresponding control cells. Therefore indicating that, at least in these conditions, IFI27 does not significantly impact the levels of PKR expression, whereas it activates PKR activation. This new explanation, has been introduced in the discussion section.

4. While the authors tested IFI27's role using SARS-CoV-2 and Vesicular Stomatitis Virus (VSV), explaining their rationale for selecting these specific viruses over others would add context and strengthen their conclusions. In addition, what is the rational behing using one virus or the other in some of the experiments?.

Response: Our lab is interested in analyzing SARS-CoV-2-host interactions. In addition, and to broaden our results on PKR activation, we have introduced an additional virus known to induce PKR activation, such as VSV.

5. In figure 2, the authors use only one time point (24 hpi) for SARS-CoV2 infections, while for VSV, the authors use two time points (8 and 24 hpi). Please explain.

Response: According to this reviewer comment, and the comment 5 from reviewer 2, we have performed additional experiments by including additional time points for SARS-CoV-2 (new Figures 3C and 4D).

marta.lopez@cnb.csic.es

Campus Universidad Autónoma de Madrid
Darwin 3, Cantoblanco, 28049 Madrid, Spain.
Tel.: +34 91 5855307
