# Supplementary material for: IFN alpha inducible protein 27 (IFI27) acts as a positive regulator of PACT-dependent PKR activation after RNA virus infections
Source: PLoS Pathog. 2025 Jun 16;21(6):e1013246. doi: 10.1371/journal.ppat.1013246 (PMC12204625; doi:10.1371/journal.ppat.1013246)
Supplement: Attachment — Submitted filename: renamed_5fe42.docx [file ppat.1013246.s012.docx]

Dr Marta L. DeDiego,

Department of Molecular and Cellular Biology

Centro Nacional de Biotecnología - CSIC,

Darwin 3, Campus de Cantoblanco,

Madrid E-28049, Spain.

Tel	(+34) 91-585-4552

Email	[marta.lopez@cnb.csic.es](mailto:marta.lopez@cnb.csic.es)

Drs. Peter Sarnow (Academic Editor), Alexander Gorbalenya (Section Editor), Sumita Bhaduri-McIntosh (Editor-in-Chief), Michael Malim (Editor-in-Chief), **Plos Pathogens**

May 21^st^, 2025

Manuscript ID number: PPATHOGENS-D-24-02606R1

Dear Editors,

Please find attached the revised version of the manuscript entitled “**IFN alpha inducible protein 27 (IFI27) acts as a positive regulator of PACT-dependent PKR activation after RNA virus infections**” by López-García *et al*., which we would like to resubmit to be further considered for publication at **Plos Pathogens*.***

We would like to thank the referees and the editors for their helpful comments, and for giving us the opportunity to revise our paper again. To address the issues raised by the reviewers, we have performed additional experiments, modified the figures and made changes to the text where appropriate. Attached you will find a complete point-by-point reply to reviewers' comments.

We think that the manuscript has been improved after the minor comments made by the referees in this second round of review, and we have included some new data. Therefore, we hope that in the light of the changes made, you will now find our manuscript suitable for publication at **Plos Pathogens**.

Looking forward to hearing from you soon, sincerely,

Marta L. DeDiego

**Point-by-point reply to the reviewers' comments**

**Part I - Summary**

**Reviewer #1**

**General comments:**

Reviewer #1: The authors have successfully addressed all the concerns that I raised last time.

Response: Thank you very much for acknowledging that we have successfully addressed the reviewer´s concerns.

**Reviewer #2**

**General comments:**

Reviewer #2: The authors addressed the concerns adequately. I have no further comments.

Response: Thank you very much for indicating that we have addressed the reviewer´s concerns adequately.

**Reviewer #3**

**General Comments:**

Reviewer #3: This manuscript explores a novel role for the interferon-stimulated gene IFI27 as a positive regulator of PKR activation through a PACT-dependent mechanism. The authors demonstrate that IFI27 interacts with both PACT and PKR in an RNA-dependent fashion, enhancing PKR phosphorylation and subsequent eIF2α phosphorylation, thereby suppressing protein translation. These effects are observed under basal conditions and during infection with RNA viruses, including SARS-CoV-2 and VSV, and are supported by a combination of proteomic analysis, co-immunoprecipitation, reporter assays, stress granule quantification, and genetic KO/KD models.
The authors have responded thoroughly to previous reviewer critiques. They provide a detailed dissection of the IFI27-PACT-PKR axis, including time- and dose-response analyses, confirmation of effects across multiple cell lines, and evidence for specificity using IFI27 mutants and rescue experiments. The revised version includes improved figure quality, better quantification, and additional experimental controls that address prior concerns about transfection bias and specificity.
The novelty lies in identifying IFI27 as a regulatory component of the PKR signaling pathway. Given the central role of PKR in translational control and innate immunity, these findings are both mechanistically important and of broad relevance to the pathogen-host interaction field. The work also opens potential avenues for therapeutic modulation of PKR activity during viral infections.
One limitation is the exclusive use of tumor-derived cell lines. While the authors acknowledge this and discuss it in the manuscript, future studies in primary cells or in vivo would be needed to fully generalize the findings. Another minor caveat is that while IFI27-PKR interactions are shown to be PACT-dependent, the RNA dependence of the IFI27-PKR complex is not directly tested with RNase treatment. Nonetheless, the presented evidence is convincing and well controlled.
Overall, the manuscript is clearly written, data-rich, and scientifically sound. It provides novel mechanistic insight into a relevant antiviral pathway and significantly improves upon the previous version.

Response: We thank the reviewer by pointing out that “The authors have responded thoroughly to previous reviewer critiques”, and that “The revised version includes improved figure quality, better quantification, and additional experimental controls that address prior concerns about transfection bias and specificity. Overall, the manuscript is clearly written, data-rich, and scientifically sound. It provides novel mechanistic insight into a relevant antiviral pathway and significantly improves upon the previous version”.

**Part II- Major Issues: Key Experiments Required for Acceptance**

**Reviewer #1**

**1.** The western blots in Fig 1D seems to be inappropriately cropped and distinct bands are not seen in some of them, bands are merged into a single line. The blots should be replaced before final submission.

Response: As suggested by the reviewer, we have performed new Western blots and replaced the Western blots in figure 1D by higher quality ones.

**Reviewer #2**

**1.**  I have no further comments

**Reviewer #3**

**1.** The study already presents a robust and novel mechanistic model, and the current dataset supports the central conclusions:
1. Clarify the RNA dependence of IFI27–PKR interaction:
The authors demonstrate that the IFI27–PACT interaction is RNA-dependent (Fig. 1B), and that IFI27–PKR binding is PACT-dependent (Fig. 9), with enhanced association upon poly(I:C) stimulation (Fig. 1E). However, RNA dependence of the IFI27–PKR complex has not been directly tested via RNase treatment. Although the authors reasonably argue that the interaction is indirect via PACT, including RNase treatment in the PKR co-IP would definitively support this model. If technically feasible, this addition would complete the mechanistic link.

Response: According to this reviewer comment, we have performed co-IP experiments using an anti-PKR antibody, in the presence of RNAses by incubating the extracts with the RNAses under two different conditions. However, unfortunately, in the less prolonged RNAse treatment, we could not detect differences in the amounts of co-immunoprecipitated IFI27 between the cell extracts not treated and treated with RNAses, likely due to an inefficient treatment to degrade the RNAs. Contrary, in the more prolonged treatment we could not detect even PKR after the immunoprecipitation in the presence of RNAses, invalidating the immunoprecipitation results. Please, note that as the reviewer indicates, we have demonstrated that the IFI27–PACT interaction is RNA-dependent (Fig. 1B), that IFI27–PKR binding is PACT-dependent (Fig. 9), and that the interaction between IFI27 and PKR is enhanced upon poly(I:C) stimulation (Fig. 1E). In addition, we do not claim a direct interaction between IFI27 and PKR. Therefore, and to avoid delaying our response to the reviewers in this second round of reviews, although we respectfully agree with the reviewer´s comment, we would prefer, if considered reasonable, to leave the manuscript as it stands now.

**Part III – Minor Issues: Editorial and Data Presentation Modifications**

**Reviewer #1:** None

**Reviewer #2:** I have no further comments

**Reviewer #3:**

1. The discussion would benefit from slightly tightening the contrasting roles of IFI27 on IFN signaling vs. PKR activation—especially to help readers reconcile the previous immunosuppressive roles with its proposed PKR activation.

Response: According to this reviewer´s comment, we have clarified in the discussion section that: “Previously, we showed that IFI27 negatively modulates the innate immune responses induced after viral infections, by modulating RIG-I and MDA5 activation (Villamayor et al. 2023; Rivero et al. 2024). In addition, in this work we show how IFI27 positively affects PKR activation. Interestingly, PKR activation could modulate the innate immune responses after viral infections by blocking the translation of antiviral and inflammatory proteins. In this sense, the decreased PKR activation in cells knocked-in or knocked-out for IFI27 could be contributing to an increased translation of innate immune response proteins, providing, therefore, an additional molecular mechanism to the effect of IFI27 in negatively modulating innate immune responses, as we previously published (Villamayor et al., 2023; Rivero et al., 2024).

2. In the methods section, the RNAbindRPlus prediction of RNA-binding sites in IFI27 could be briefly described with confidence scores or thresholds.

Response: Following this referee´s comment, we have introduced in the results section the following information: “IFI27 contains 13 amino acids which are predicted to bind RNA, based on score predictions higher than 0.5. These IFI27 amino acids predicted to bind RNA and their predicted scores are as follows: amino acid 60 (0.68), 61 (0.61), 62 (0.60), 63 (0.53), 64 (0.70), 65 (0.69), 68 (0.64), 69 (0.65), 82 (0.53), 83 (0.58), 84 (0.60), 85 (0.61) and 86 (0.62). In contrast, the predicted scores for the other residues were below 0.1 for 90% of them, and below 0.41 for all of them”.

3. Consider standardizing the naming of IFI27 mutants (e.g., IFI27-S63L vs. S63L variant) for clarity.

Response: Taking into consideration this comment, we have standardized the naming of S63L and V82A variants through the manuscript.

4. The IFI27-PACT interaction remains inferred as RNA-dependent but would benefit from RNase treatment during PKR co-IPs, not just PACT co-IPs. Nonetheless, the authors do not claim a direct interaction and have added poly(I:C)-dependent modulation data, which strengthens their model.

Response: According to this reviewer comment, and as indicated in comment 1 (part II), of this same reviewer, we have performed co-IP experiments using an anti-PKR antibody, in the presence of RNAses by incubating the extracts with the RNAses under two different conditions. However, unfortunately, in the less prolonged RNAse treatment, we could not detect differences in the amounts of co-immunoprecipitated IFI27 between the cell extracts not treated and treated with RNAses, likely due to an inefficient treatment to degrade the RNAs. Conversely, in the more prolonged treatment we could not detect even PKR after the immunoprecipitation in the presence of RNAses, invalidating the immunoprecipitation results. Please, note that we have demonstrated that the IFI27–PACT interaction is RNA-dependent (Fig. 1B), that the binding of IFI27 to PKR is PACT-dependent (Fig. 9), and that the interaction between IFI27 and PKR is enhanced upon poly(I:C) stimulation (Fig. 1E). In addition, we do not claim a direct interaction between IFI27 and PKR. Therefore, and to avoid delaying our response to the reviewers in this second round of reviews, although we agree with the reviewer´s comment that these experiments would strengthen our model, we would prefer, if possible, to leave the manuscript as it stands now.
